# Prolonged seizure activity causes caspase dependent cleavage and dysfunction of G-protein activated inwardly rectifying potassium channels

**DOI:** 10.1038/s41598-017-12508-y

**Published:** 2017-09-26

**Authors:** Brian C. Baculis, Amanda C. Weiss, Weilun Pang, Han Gil Jeong, Jun Hee Lee, Dai-Chi Liu, Nien-Pei Tsai, Hee Jung Chung

**Affiliations:** 1 Department of Molecular and Integrative Physiology, University of Illinois at Urbana-Champaign, Urbana, Illinois 61801 USA; 2 Neuroscience Program, University of Illinois at Urbana-Champaign, Urbana, Illinois 61801 USA

## Abstract

Recurrent high-frequency epileptic seizures cause progressive hippocampal sclerosis, which is associated with caspase-3 activation and NMDA receptor-dependent excitotoxicity. However, the identity of caspase-3 substrates that contribute to seizure-induced hippocampal atrophy remains largely unknown. Here, we show that prolonged high-frequency epileptiform discharges in cultured hippocampal neurons leads to caspase-dependent cleavage of GIRK1 and GIRK2, the major subunits of neuronal G protein-activated inwardly rectifying potassium (GIRK) channels that mediate membrane hyperpolarization and synaptic inhibition in the brain. We have identified caspase-3 cleavage sites in GIRK1 (^387^ECLD^390^) and GIRK2 (^349^YEVD^352^). The YEVD motif is highly conserved in GIRK2-4, and located within their C-terminal binding sites for Gβγ proteins that mediate membrane-delimited GIRK activation. Indeed, the cleaved GIRK2 displays reduced binding to Gβγ and cannot coassemble with GIRK1. Loss of an ER export motif upon cleavage of GIRK2 abolishes surface and current expression of GIRK2 homotetramic channels. Lastly, kainate-induced status epilepticus causes GIRK1 and GIRK2 cleavage in the hippocampus *in vivo*. Our findings are the first to show direct cleavage of GIRK1 and GIRK2 subunits by caspase-3, and suggest the possible role of caspase-3 mediated down-regulation of GIRK channel function and expression in hippocampal neuronal injury during prolonged epileptic seizures.

## Introduction

Epilepsy is a chronic brain disorder characterized by recurrent epileptic seizures indicative of neuronal hyperexcitability^1^. Temporal lobe epilepsy (TLE) is the most common epilepsy in adults^2^ and is associated with progressive hippocampal sclerosis, cognitive decline, and drug-resistant seizures^2–4^. Studies in human TLE and kainate-induced rodent models of TLE suggest that both excitotoxicity induced by excessive activation of N-methyl-D-aspartate receptors (NMDARs) and apoptotic mechanisms including activation of cysteinyl aspartate-specific protease (caspase) family proteins may contribute to hippocampal atrophy following chronic epileptic seizures^3,5^. However, the identity of caspase substrates that contribute to seizure-induced neuronal death remains largely unknown.

G protein-activated inwardly rectifying potassium (GIRK) channels belong to the Kir3.x subfamily of inwardly rectifying potassium channels, which potently inhibit neuronal excitability^6^. Most neuronal GIRK channels are heterotetramers of GIRK1 and GIRK2 subunits^7^, and to a lesser extent heterotetramers of GIRK3 and GIRK2^8^, although some neuronal populations contain GIRK2a or GIRK2c homotetrameric channels^9^, or GIRK4^10^. Neuronal GIRK channels are preferentially localized in the soma and dendrites of hippocampal neurons^11,12^ where they mediate hyperpolarization induced by activation of adenosine A_1_ and GABA_B_ receptors^13,14^ and give rise to slow inhibitory postsynaptic current upon GABA_B_ receptor activation^15^. Importantly, mice deficient in GIRK2 display decreased GIRK1 protein expression, sporadic seizures, increased susceptibility to the convulsant agents, and a shortened lifespan due to spontaneous lethal seizures^16^, suggesting that the loss of GIRK channel function contribute to pathologic hyperexcitability. In contrast, GIRK1 agonists delay seizure onset, prevent convulsions, and reduce lethality in animal models of epilepsy^17^, further confirming the protective role of GIRK channels against seizures.

We have previously reported that NMDARs can be activated by synaptically released glutamate in cultured hippocampal neurons upon removal of the chronic pharmacological NMDAR blockade in the absence of magnesium (Mg^2+^)^18^. Such activation of synaptic NMDARs causes high frequency burst discharges and increases surface expression of GIRK1 and GIRK2 within 15 min, basal GIRK current, and GIRK activation by adenosine A_1_ receptors^18,19^, suggesting that this initial increase in GIRK surface density may serve as a homeostatic response to dampen membrane excitability.

In this study, we discovered that prolonged epileptiform seizure activity in cultured primary rat hippocampal neurons for >30 min resulted in caspase dependent cleavage of surface and intracellular GIRK1 and GIRK2 subunits in their cytoplasmic C-terminal tails. Similarly, kainate-induced status epilepticus in rats also induced C-terminal cleavage of GIRK1 and GIRK2 in their hippocampi. We found that caspase-3 directly cleaved GIRK1 at ^387^ECLD^390^ and GIRK2 at ^349^YEVD^352^. Mutant GIRK2-Y353X, which mimics truncated GIRK2 upon caspase-3 mediated cleavage, displayed reduced binding to Gβγ and GIRK1. Furthermore, truncated GIRK2-Y353X channels failed to express on the plasma membrane and display K^+^ current. Given that hippocampal neuronal apoptosis induced by prolonged high-frequency epileptiform discharges is mediated by activation of NMDARs and caspase-3 family proteins^20,21^, our data suggest that this novel down-regulation of GIRK channels by caspase-3 may contribute to NMDAR-dependent hippocampal atrophy following chronic epileptic seizures.

## Results

### Caspase-3 induces C-terminal cleavage of GIRK channels in hippocampal neurons upon prolonged seizure activity

**Figure 1:**
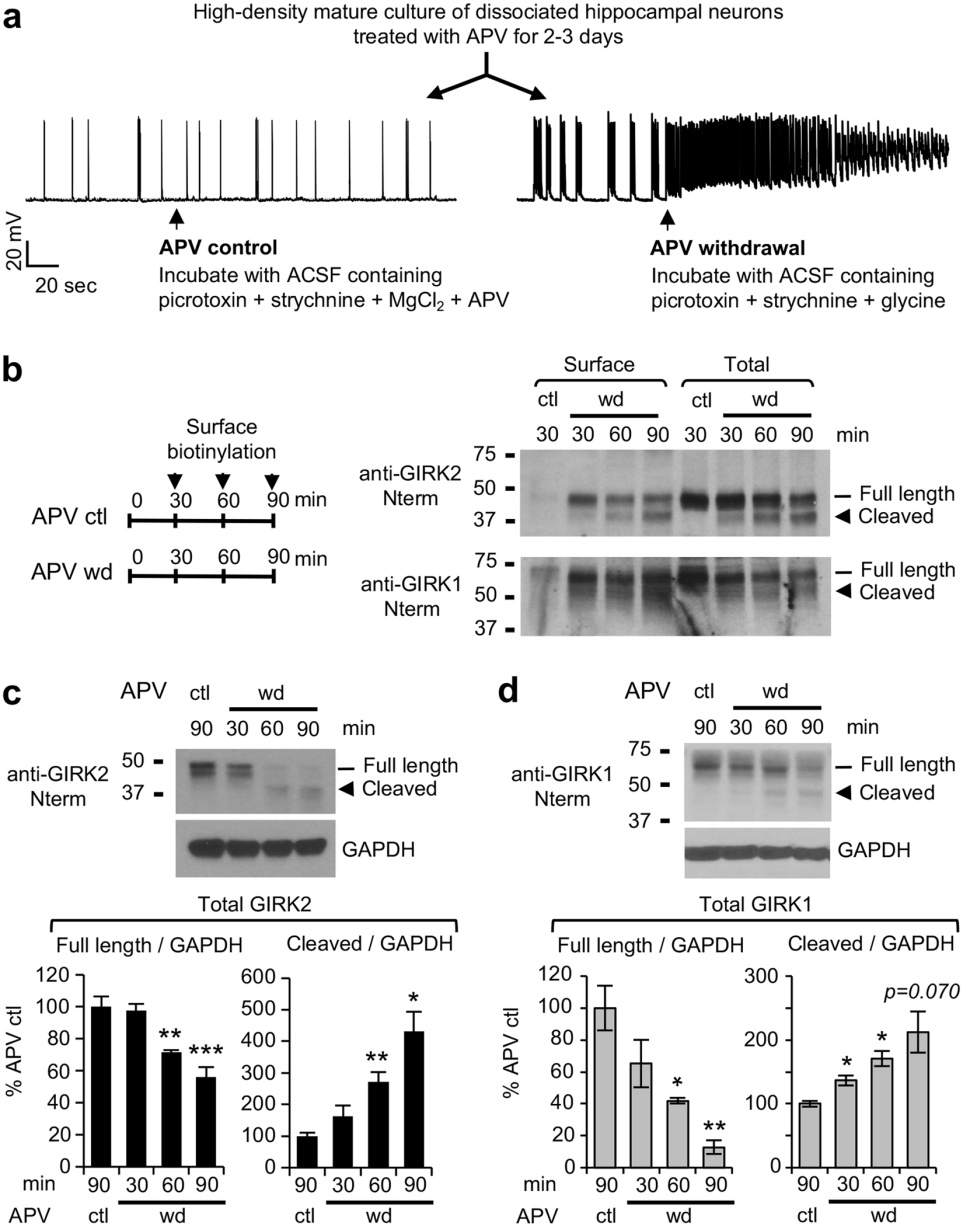
Prolonged seizure activity induces C-terminal cleavage of GIRK channels in cultured hippocampal neurons. (**a**) Whole-cell patch clamp recording of spontaneous action potentials in cultured hippocampal neurons (12–13 DIV, pretreated with 200 μM DL-APV for 2–3 days at 10–11 DIV) before and after APV control (left representative trace) and APV withdrawal (right representative trace). Activation of synaptic NMDAR upon APV withdrawal induced high frequency burst firing of action potentials and sustained depolarization. (**b**) Surface biotinylation of cultured hippocampal neurons after APV control (ctl) or APV withdrawal (wd) was analyzed by immunoblotting with antibodies recognizing intracellular N-termini of GIRK1 and GIRK2. Prolonged APV withdrawal for 30–90 min resulted in the C-terminal cleavage of GIRK1 and GIRK2 proteins that were biotinylated (Surface) and in the lysates (Total). (**c**,**d**) Quantitative immunoblot analyses of total GIRK2 (**c**) and GIRK1 (**d**) expression in cultured hippocampal neurons after APV control for 90 min (ctl, n = 4–5) or APV withdrawal (wd) for 30–90 min (n = 3–4 each time point). GAPDH served as a loading control. Data shown represent the mean ± SEM. *p < 0.05, **p < 0.01, ***p < 0.005. The cropped gray-scale blots are displayed. Full-length blots are included in the Supplementary Fig. S3.

To test whether prolonged epileptic seizures can regulate GIRK channels, we first treated high-density rat hippocampal neuronal culture with the NMDAR antagonist 2-amino-5-phosphonovaleric acid (APV) for 2-3 days at 10–11 days *in vitro* (DIV) when mature synapses appear^22^. Then the cultured neurons were incubated in fresh artificial cerebrospinal fluid (ACSF) bath solution containing NMDAR co-agonist glycine, GABA_A_ receptor antagonist picrotoxin, glycine receptor antagonist strychnine, but not Mg^2+^ and APV^18,19^ (Fig. 1a). Such activation of NMDAR by glycine and synaptically released glutamate upon APV withdrawal induced high-frequency burst firing of action potentials and sustained depolarization (Fig. 1a), similar to those seen in well-established hippocampal neuronal culture models for epileptic seizure activity^20,23–26^, as well as epileptic seizures seen *in vivo* ^27,28^. This APV withdrawal treatment increased action potential firing rates and burst firing which lasted up to 90 min (Supplementary Fig. S1). In contrast, neurons incubated in fresh ACSF solution containing APV and Mg^2+^ (APV control) displayed occasional spontaneous burst firing but not high-frequency epileptiform discharges in the presence of picrotoxin and strychnine (Fig. 1a).

Surface biotinylation revealed that APV withdrawal increased surface expression of endogenous GIRK1 and GIRK2 within 30 min (Fig. 1b), consistent with our previous report demonstrating a similar increase within 15 min^18^. Surprisingly, APV withdrawal over 30–90 min led to a progressive increase in truncated GIRK1 and GIRK2 proteins on the plasma membrane and total lysate while decreasing surface and total expression of full length proteins (Fig. 1b–d). While smears or ladders of truncated GIRK1 proteins from 50 kD band were detected upon prolonged APV withdrawal, a distinct immunoblot band of truncated GIRK2 proteins were observed around 40 kD (Fig. 1b–d). This cleavage occurred in their C-terminal domains because GIRK subunits were immunoblotted with antibodies that recognize their intracellular N-termini (Fig. 1b–d, Supplementary Fig. S2).

**Figure 2:**
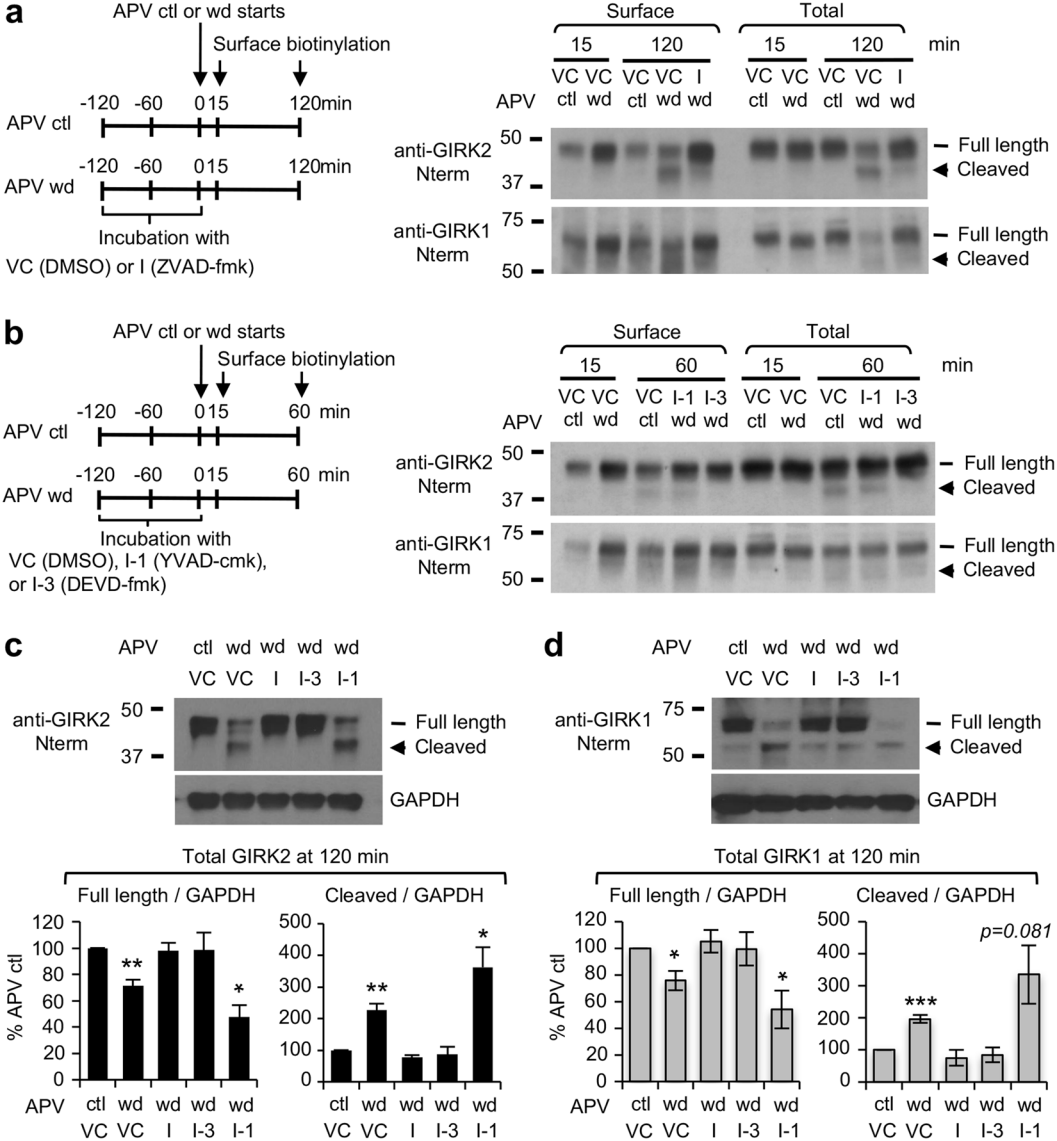
Caspase-3 mediates C-terminal cleavage of GIRK channels in cultured hippocampal neurons upon prolonged seizure activity. (**a**,**b**) Surface biotinylation was performed after APV control (ctl) or APV withdrawal (wd) in cultured hippocampal neurons pretreated for 2 h with vehicle control (VC, 0.1% DMSO), pan caspase inhibitor ZVAD-Fmk (I, 100 μM) (**a**), caspase-1 inhibitor YVAD-cmk (I-1, 20 μM), or caspase-3 inhibitor DEVD-fmk (I-3, 20 μM) (**b**). Surface and Total GIRK1 and GIRK2 proteins were examined by immunoblotting with antibodies recognizing intracellular N-termini of GIRK1 and GIRK2. (**c**,**d**) Quantitative immunoblot analyses of total GIRK2 (**c**) and GIRK1 (**d**) expression after APV control (ctl) or APV withdrawal (wd) for 120 min in cultured hippocampal neurons which were pretreated for 2 h with vehicle control (VC), ZVAD-Fmk (I), YVAD-cmk (I-1), or DEVD-fmk (I-3). GAPDH served as a loading control. Pretreatment with ZVAD-Fmk and DEVD-fmk but not YVAD-cmk blocked C-terminal cleavages of GIRK1 and GIRK2 induced by prolonged APV withdrawal. Data shown represent the mean ± SEM (n = 4 per treatment). *p < 0.05, **p < 0.01, ***p < 0.005. The cropped gray-scale blots are displayed. Full-length blots are included in the Supplementary Fig. S4.

Induction of high-frequency epileptic seizure activity in cultured hippocampal neurons causes activation of caspases and apoptosis^20^. Compared to APV control-treated neurons which displayed high levels of full-length GIRK1 and GIRK2 and low levels of truncated subunits, APV withdrawal for 60 min and 120 min significantly decreased the levels of full-length subunits, and increased the levels of truncated subunits (Fig. 2a–d). This C-terminal cleavage of surface and total GIRK1 and GIRK2 was blocked by pretreatment with cell-permeable irreversible pan-caspase inhibitor ZVAD-fmk (Fig. 2a,c,d). To identify which caspases were responsible for GIRK1 and GIRK2 cleavage, we repeated the experiments with a widely used caspase-3 inhibitor DEVD-fmk that also inhibits caspase-6, 7, 8 and 10. Pretreatment with DEVD-fmk blocked the C-terminal cleavage of GIRK subunits and inhibited the reduction in the level of full-length subunits induced by prolonged APV withdrawal (Fig. 2b–d). In contrast, seizure activity-induced cleavage of GIRK subunits was unaffected by pretreatment with YVAD-cmk that inhibits caspase-1, 4 and 5 (Fig. 2b–d).

### Caspase-3 directly cleaves GIRK1, GIRK2, and GIRK4 *in vitro*

**Figure 3:**
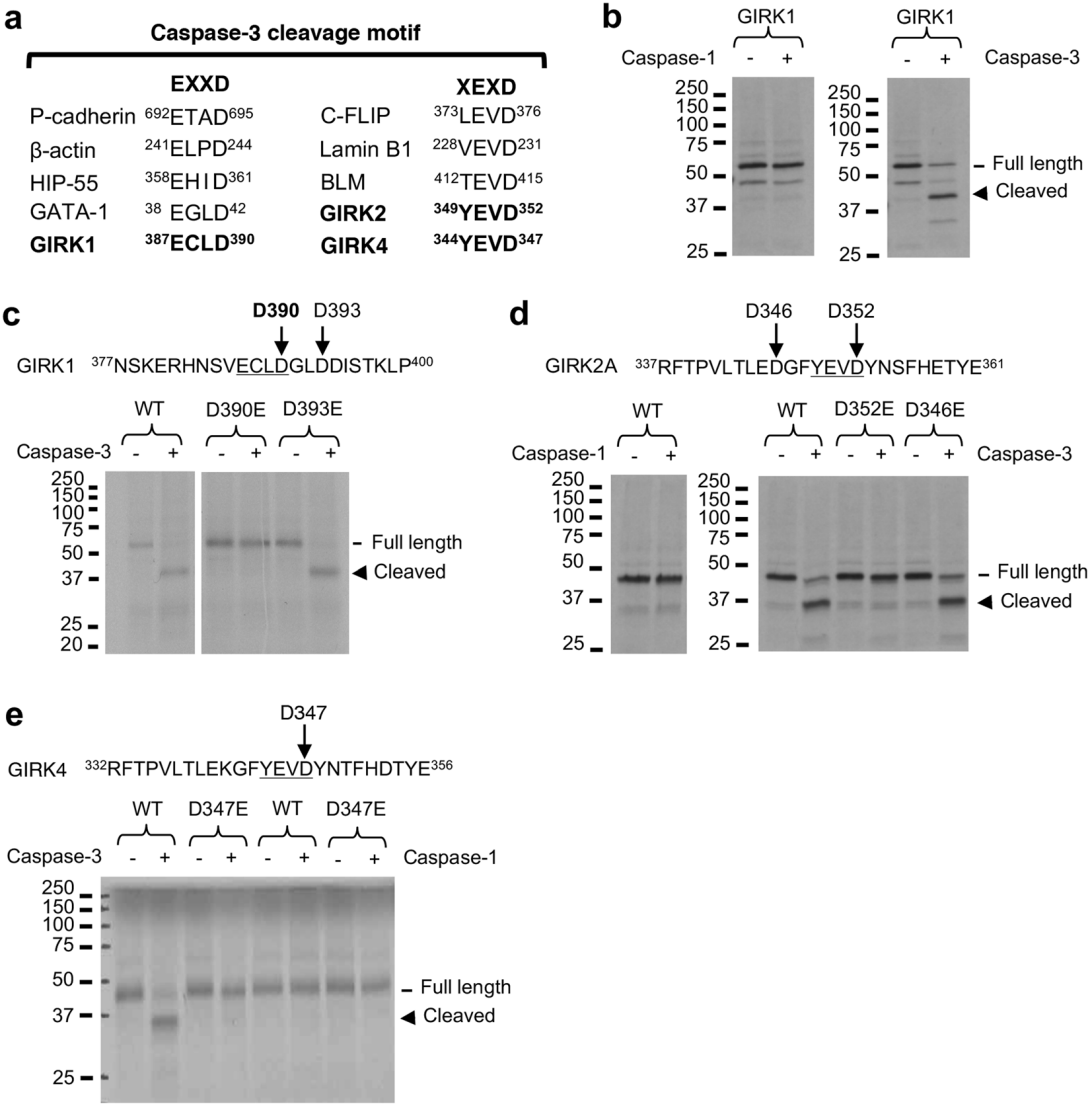
Caspase-3 directly cleaves GIRK1, GIRK2, and GIRK4 *in vitro*. (**a**) Sequence alignment of GIRK subunits with caspase-3 cleavage sites of known substrates that resemble consensus EXXD and XEXD motifs. (**b**–**e**) *In vitro* transcription and translation of GIRK subunits with ^35^S–methionine followed by *in vitro* cleavage assay with purified caspase-1 or caspase-3. The cleavage reaction products were separated by SDS-PAGE gel and visualized by autoradiograph. (**b**) Caspase-3 but not caspase-1 cleaved wild type (WT) GIRK1. (**c**) Caspase-3 also cleaved mutant GIRK1-D393E but not mutant GIRK1-D390E, indicating that D390 within ^387^ECLD^390^ motif is the caspase-3 cleavage site. (**d**) Caspase-3 but not caspase-1 cleaved GIRK2 WT. Caspase-3 also cleaved mutant GIRK2-D346E but not mutant GIRK2-D352E, indicating that D352 within ^349^YEVD^352^ motif is the caspase-3 cleavage site. (**e**) While caspase-1 had no effect, caspase-3 cleaved GIRK4 WT but not mutant GIRK4-D347E, indicating that D347 within ^344^YEVD^347^ motif is the caspase-3 cleavage site.

Our results from Fig. 2b–d suggest that GIRK1 and GIRK2 cleavage upon prolonged seizure activity may likely be mediated by caspases which cleave their substrates at DEVD, EXXD, or XEXD motifs after aspartate (D) residue^29,30^. One of the primary caspases in this family is caspase-3, which is the main executioner of apoptosis^29,30^ and has been implicated in hippocampal sclerosis after epileptiform activity^20,21^. Sequence alignment of GIRK subunits with known caspase-3 substrates revealed the presence of ^387^ECLD^390^ in GIRK1 similar to EXXD motif^29,30^, and YEVD motifs in GIRK2 and GIRK4 that matched XEXD motif (Fig. 3a). *In vitro* cleavage assay with purified caspases revealed that caspase-3 cleaved GIRK1 and GIRK2A while capsase-1 did not (Fig. 3b,d). These results are consistent with our previous results that caspase-3 inhibitor DEVD-fmk but not caspase-1 inhibitor YVAD-cmk, blocked seizure-induced GIRK1 and GIRK2 cleavage in cultured hippocampal neurons (Fig. 2b–d).

Caspases cleave substrates with 20,000-fold preference for aspartate over glutamate (E) residue^31^. Caspase-3 failed to cleave mutant GIRK1 in which the aspartate residue in ^387^ECLD^390^ is mutated to glutamate (D390E) but not the GIRK1 containing D393E mutation (Fig. 3c). Caspase-3 cleaved mutant GIRK2A containing D346E mutation located upstream of ^349^YEVD^352^ motif but not GIRK2A harboring D352E mutation within this motif (Fig. 3d). Similarly, D347E mutation in ^344^YEVD^347^ motif of GIRK4 abolished caspase-3 mediated cleavage of GIRK4 (Fig. 3e). Although recent publication has shown that caspases can cleave at glutamate residue with slower kinetics in synthetic peptide substrates^31^, a complete absence of caspase-3 mediated cleavage of GIRK1-D390E, GIRK2-D352E, and GIRK4-D347E in 90 min reaction indicates that ^387^ECLD^390^ of GIRK1, ^349^YEVD^352^ in GIRK2, and ^344^YEVD^347^ in GIRK4 are caspase-3 cleavage motifs.

### GIRK2A truncated at ^349^YEVD^352^ motif shows decreased binding to Gβγ and GIRK1

**Figure 4:**
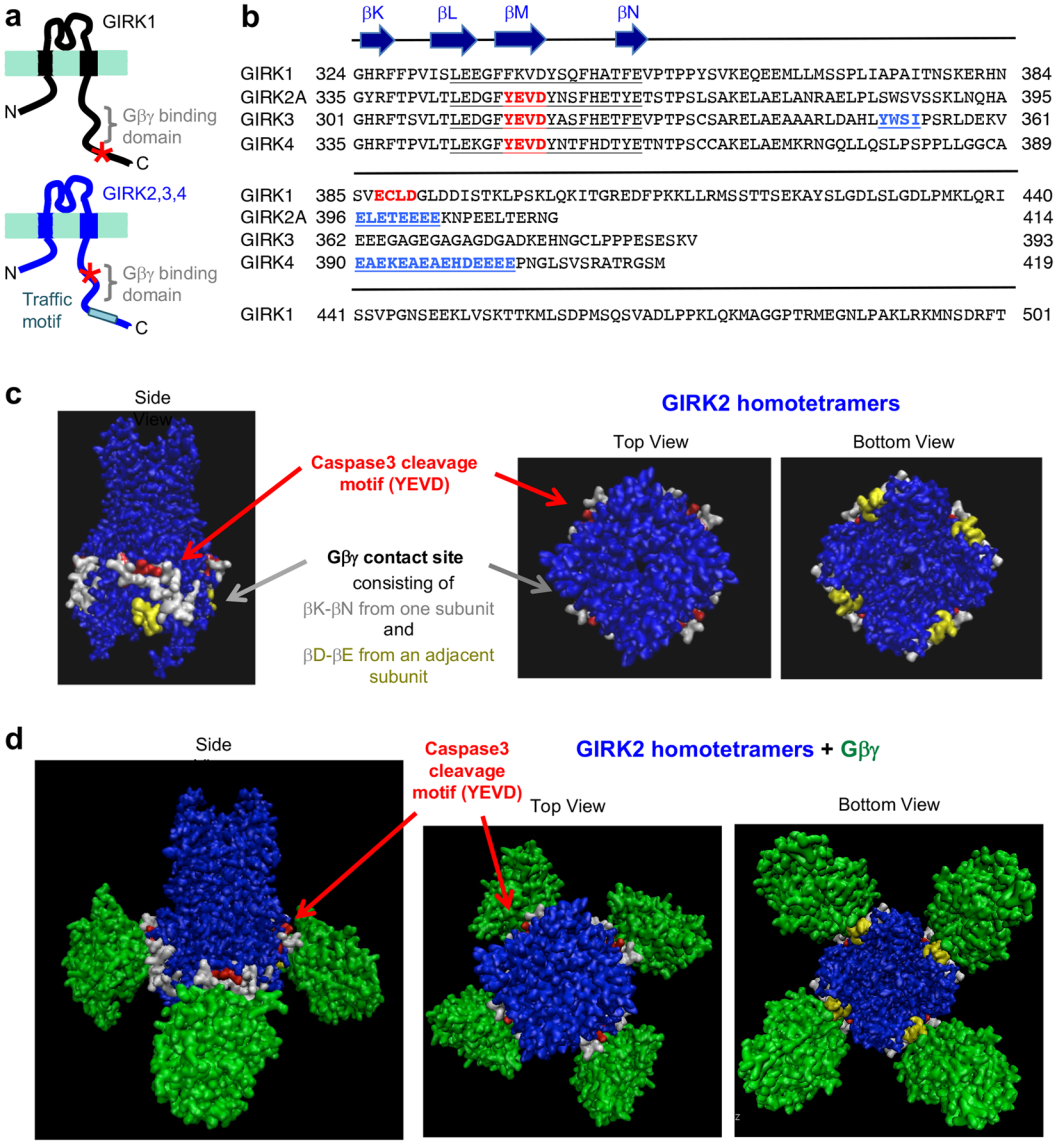
Caspase-3 cleavage motif is located at the Gβγ contact site of GIRK2. (**a**) Schematic cartoons (not to scale) of GIRK1 and GIRK2 subunit, highlighting the Gβγ binding site and an ER export motif in their cytoplasmic C-terminal domains. *Indicates caspase-3 cleavage motifs. (**b**) Amino acid sequence alignment of GIRK1 (NP_113798.1), GIRK2A (NP_001020755.1), GIRK3 (NP_032455.2), and GIRK4 (NP_058993.1) using BLAST (http://www.ncbi.nlm.nih.gov/BLAST/). Caspase-3 cleavage sites in GIRK1 (^387^ECLD^390^), GIRK2A (^349^YEVD^352^), GIRK3 (^315^YEVD^318^), and GIRK4 (^344^YEVD^347^) are shown in red. The traffic motifs are shown in light blue. The Gβγ contact sites are underlined. (**c**,**d**) Molecular surface representation of the crystal structure of GIRK2 homotetrameric channels alone (**c**, shown in blue) or complexed with Gβγ (**d**, shown in green) viewed from the side, top, and bottom (Protein Data Bank (PDB): 4kfm). Residues in red show caspase-3 cleavage site of GIRK2 (^349^YEVD^352^). Residues in yellow and white show the Gβγ contact site of GIRK2.

GIRK channels are gated by direct binding of Gβγ in response to neurotransmitters and neuromodulators that activate G protein-coupled receptors (GPCR) coupled to pertussis toxin-sensitive Gi/o proteins^32,33^. GIRK2 homotetrameric channels possess four Gβγ binding sites at the well-conserved cytoplasmic interfaces between adjacent subunits that contribute to formation of the extended cytoplasmic pore^34^. Specifically, the Gβγ contact site in GIRK2 is formed by secondary β sheet structure elements βK, βL, βM and βN from one subunit and by elements βD and βE from an adjacent subunit^34^. Sequence alignment of the distal C-terminal tails of all GIRK subunits revealed that caspase-3 cleavage site ^387^ECLD^390^ of GIRK1 is located distal to its Gβγ contact site whereas caspase-3 cleavage sites (YEVD) of GIRK2, GIRK3, and GIRK4 are located at their secondary βM sheet structure elements within the Gβγ contact site (Fig. 4a,b).

**Figure 5:**
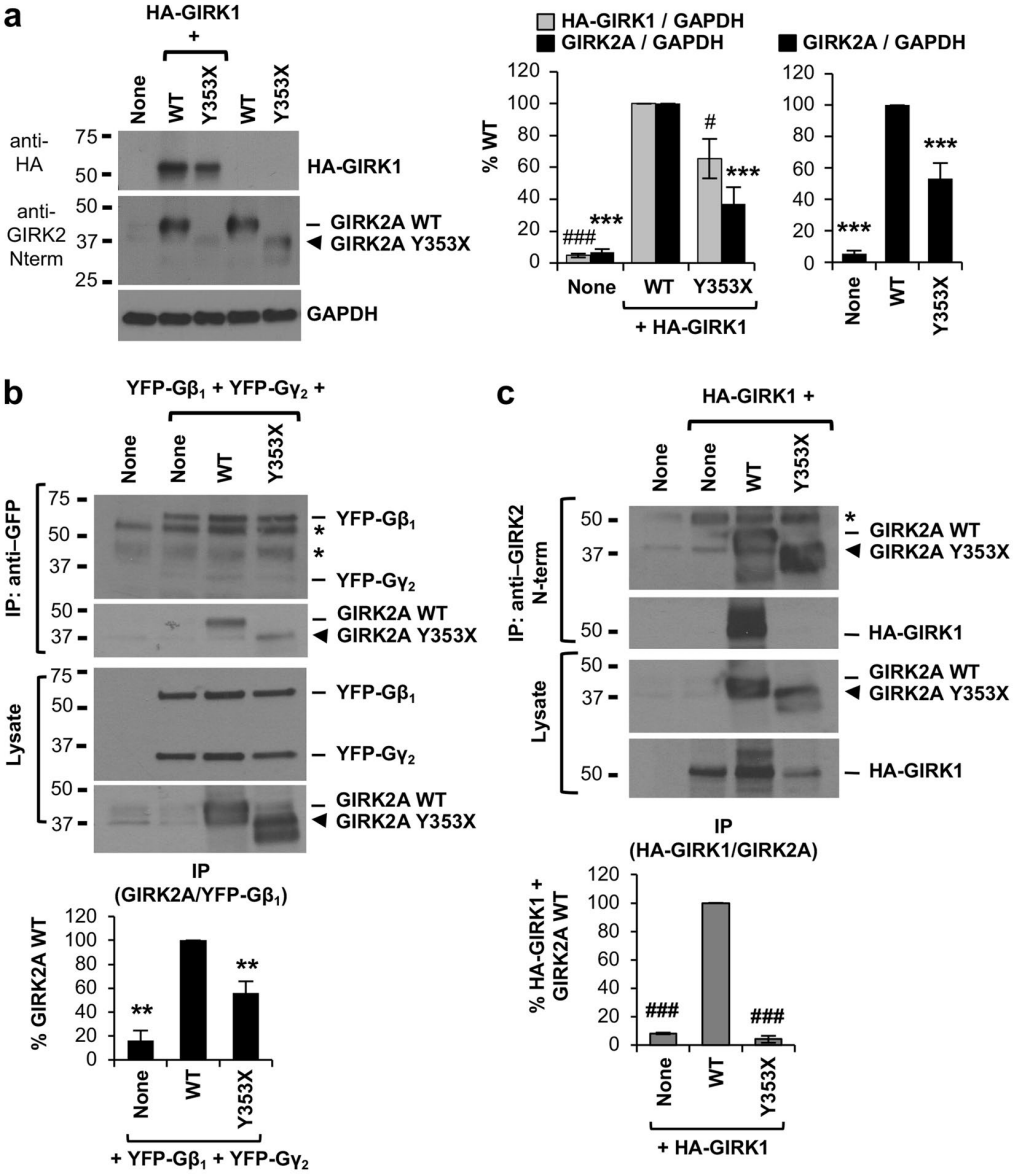
GIRK2A truncated at ^349^YEVD^352^ motif shows decreased binding to Gβγ and GIRK1. (**a**) Quantitative immunoblot analyses of the HEK293T cells transfected with equal amounts of plasmids containing GIRK1 with extracellular HA tag (HA-GIRK1) and GIRK2A wild type (WT) or GIRK2A-Y353X (n = 7–8 each), GIRK2A WT alone, or GIRK2A-Y353X alone (n = 6–7 each). GAPDH served as a loading control. GIRK2A-Y353X expression was significantly lower than GIRK2A WT expression. Coexpression of GIRK2A-Y353X decreased HA-GIRK1 expression compared to coexpression of GIRK2A WT. (**b**) Immunoprecipitation (IP) with anti-GFP antibodies was performed from untransfected HEK293T cells (none, n = 4), or the cells transfected with YFP-Gβ_1_ and YFP-Gγ_2_ alone (n = 4) or together with GIRK2A WT (n = 6) or GIRK2A-Y353X (n = 6). To increase GIRK2A-Y353X expression to a similar extent as GIRK2A-WT expression, we doubled the amount of GIRK2A-Y353X plasmid for transfection compared to GIRK2A-WT plasmids. Coimmunoprecipitation of GIRK2-Y353X with Gβ_1_γ_2_ was decreased compared to GIRK2A WT. (**c**) IP with anti-GIRK2 N-terminal antibodies was performed from untransfected HEK293T cells (none), or the cells expressing HA-GIRK1 alone or together with GIRK2A WT or GIRK2A-Y353X (n = 3 each). To increase GIRK2A-Y353X expression to a similar extent as GIRK2AWT expression, we doubled the amount of GIRK2A-Y353X plasmid for transfection compared to GIRK2A-WT plasmids. Coimmunoprecipitation of HA-GIRK1 with GIRK2A-Y353X was reduced compared to GIRK2A WT. In (**b**,**c**), *points at IgG bands. Data shown represent the mean ± SEM (**p < 0.01, ***p < 0.005 against GIRK2A WT; ^#^ *p* < 0.05, ^###^ *p* < 0.005 against HA-GIRK1 + GIRK2A WT). The cropped gray-scale blots are displayed. Full-length blots are included in the Supplementary Fig. S5.

Since ^349^YEVD^352^ motif of GIRK2 falls within and near the Gβγ contact sites (Fig. 4c,d), caspase-3 cleavage of GIRK2 may disrupt GIRK2 interaction with Gβγ. To test this, co-immunoprecipitation of Gβγ was performed with wild type GIRK2A or truncated GIRK2A-Y353X in which Y353 residue right after ^349^YEVD^352^ motif was mutated to a stop codon. Thus, GIRK2A-Y353X mimics GIRK2A cleaved by caspase-3. We first found that the level of GIRK2A-Y353X proteins was consistently lower compared to wild type GIRK2A proteins although an equal amount of each plasmid was transfected in HEK293T cells (Fig. 5a). To increase the expression level of GIRK2A-Y353X to a similar extent as wild type GIRK2A for coimmunoprecipitation, we doubled the amount of GIRK2A-Y353X plasmid for transfection compared to wild type GIRK2A plasmid (Fig. 5b). Immunoprecipitation of Gβ_1_γ_2_ tagged with yellow fluorescent proteins (YFP) resulted in coimmunoprecipitation of wild type GIRK2A (Fig. 5b), consistent with the previous reports demonstrating their interactions^34,35^. In contrast, coimmunoprecipitation of GIRK2A-Y353X with YFP-Gβ_1_ and YFP-Gγ_2_ was decreased to 50% of wild-type GIRK2A (Fig. 5b), indicating that truncated GIRK2A-Y353X showed decreased binding to Gβγ.

We also noticed a significant decrease in GIRK1 expression when this subunit is coexpressed with GIRK2A-Y353X compared to wild type GIRK2A (Fig. 5a). Since GIRK1 heteromerization with GIRK2 stabilizes GIRK1 expression^36,37^, we hypothesized that caspase-3 cleavage of GIRK2 may disrupt GIRK2 interaction with GIRK1. To test this, we first doubled the amount of GIRK2A-Y353X plasmid for transfection compared to wild type GIRK2A plasmids, and performed immunoprecipitation using the same amount of anti-GIRK2 N-terminal antibodies that could immunoprecipitate a fraction but not all of transfected GIRK2A subunits from the lysate. Although the same amount of wild type GIRK2A and GIRK2A-Y353X proteins were immunoprecipitated, GIRK1 co-immunoprecipitated only with wild type GIRK2A but not GIRK2A-Y353X (Fig. 5c), indicating that truncated GIRK2A-Y353X could not coassemble with GIRK1.

### GIRK2A truncated at ^349^YEVD^352^ motif does not express at the plasma membrane

**Figure 6:**
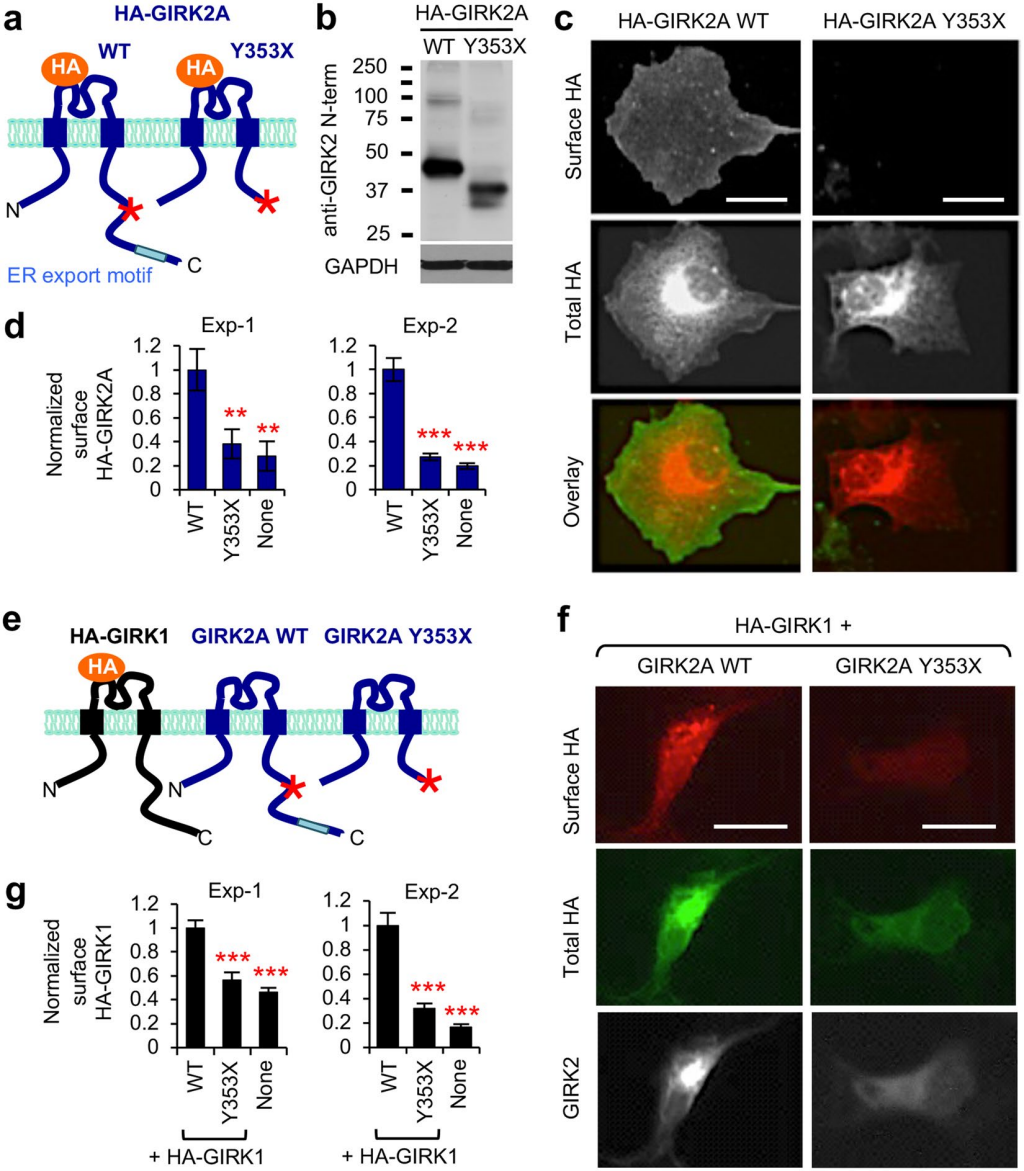
GIRK2A truncated at ^349^YEVD^352^ motif does not express at the plasma membrane. (**a**) Schematic cartoon (not to scale) of GIRK2A WT or Y353X containing extracellular HA tag (HA-GIRK2A). *Indicates caspase-3 cleavage site of GIRK2A. (**b**) Immunoblot of HA-GIRK2A WT or Y353X in transfected COS7 cells. (**c**,**d**) Surface immunostaining of COS7 cells transfected with HA-GIRK2A WT or Y353X was performed without permeabilization using mouse anti-HA antibodies and Alexa488-conjugated secondary antibodies (Exp-1, n = 10 cells per transfection) or biotin-conjugated secondary antibodies followed by Alexa488-conjugated streptavidin (Exp-2, n = 12–15 cells per transfection). Following fixation and permeabilization, their total (surface and intracellular) expression was visualized with rabbit anti-HA antibodies and Alexa594-conjugated secondary antibodies. (**c**) Representative images showing low level of HA-GIRK2A Y353X at the plasma membrane compared to WT channels. Scale bars are 15 μm. (**d**) Background subtracted, mean fluorescence intensities of surface HA in untransfected COS7 cells (none) or cells expressing HA-GIRK2A WT or Y353X. (**e**) Schematic cartoon (not to scale) of GIRK2A WT, Y353X, and GIRK1 containing extracellular HA tag (HA-GIRK1). *Indicates caspase-3 cleavage site of GIRK2A. (**f**,**g**) Surface immunostaining of COS7 cells transfected with HA-GIRK1 and GIRK2A WT or Y353X was performed without permeabilization using rabbit anti-HA antibodies and Alexa594-conjugated secondary antibodies (Exp-1, n = 24–26 cells per transfection) or biotin-conjugated secondary antibodies followed by Alexa594-conjugated streptavidin (Exp-2, n = 10 cells per transfection). Following fixation and permeabilization, total HA-GIRK1 and GIRK2A proteins were labeled with mouse anti-HA and rabbit anti-GIRK2 N-term antibodies, respectively, followed by Alexa488- and Alexa-680-conjugated secondary antibodies. (**f**) Representative images showing low level of HA-GIRK1/GIRK2A Y353X channels at the plasma membrane compared to WT channels. Scale bars are 15 μm. (**g**) Background subtracted, mean fluorescence intensities of surface HA-GIRK1 in untransfected COS7 cells (none) or cells coexpressing GIRK2A WT, or Y353X. Data shown represent the mean ± SEM. **p < 0.01, ***p < 0.005.

Trafficking of GIRK channels is tightly regulated by multiple amino acid sequence motifs within their subunits that control their forward trafficking from the endoplasmic reticulum (ER) as well as post-ER endocytic trafficking^18,36–38^. GIRK1 subunits are retained in the ER when expressed alone, but GIRK1 assembly with GIRK2 containing ER export motif ^396^ELETEEE^403^ allows efficient surface expression of heterotetrameric channels^36,37^. The C-terminal tail of GIRK2 distal to its ^349^YEVD^352^ motif contains this ER export motif (Figs 4b and 6)^36^. To test if caspase-3 cleavage of GIRK2 and subsequent loss of its ER export motif disrupts surface expression of GIRK2 channels or GIRK1/GIRK2 channels, surface immunostaining was performed in COS7 cells transfected with GIRK1 or GIRK2A, which were tagged with an extracellular hemagglutinin (HA) epitope (Fig. 6a,b,e). While wild type HA-GIRK2A channels and HA-GIRK1/GIRK2A channels displayed robust surface expression, truncated HA-GIRK2A-Y353X channels or HA-GIRK1/GIRK2A-Y353X channels failed to express on the plasma membrane (Fig. 6c,d,f–g). Lower total expression of HA-GIRK1 was observed in the cells cotransfected with GIRK2A-Y353X compared to the cells expressing wild type GIRK2A (Fig. 6f), similar to our western blot analyses in HEK293T cells (Fig. 5a).

### GIRK2A channels truncated at ^349^YEVD^352^ motif do not express K^+^ current

**Figure 7:**
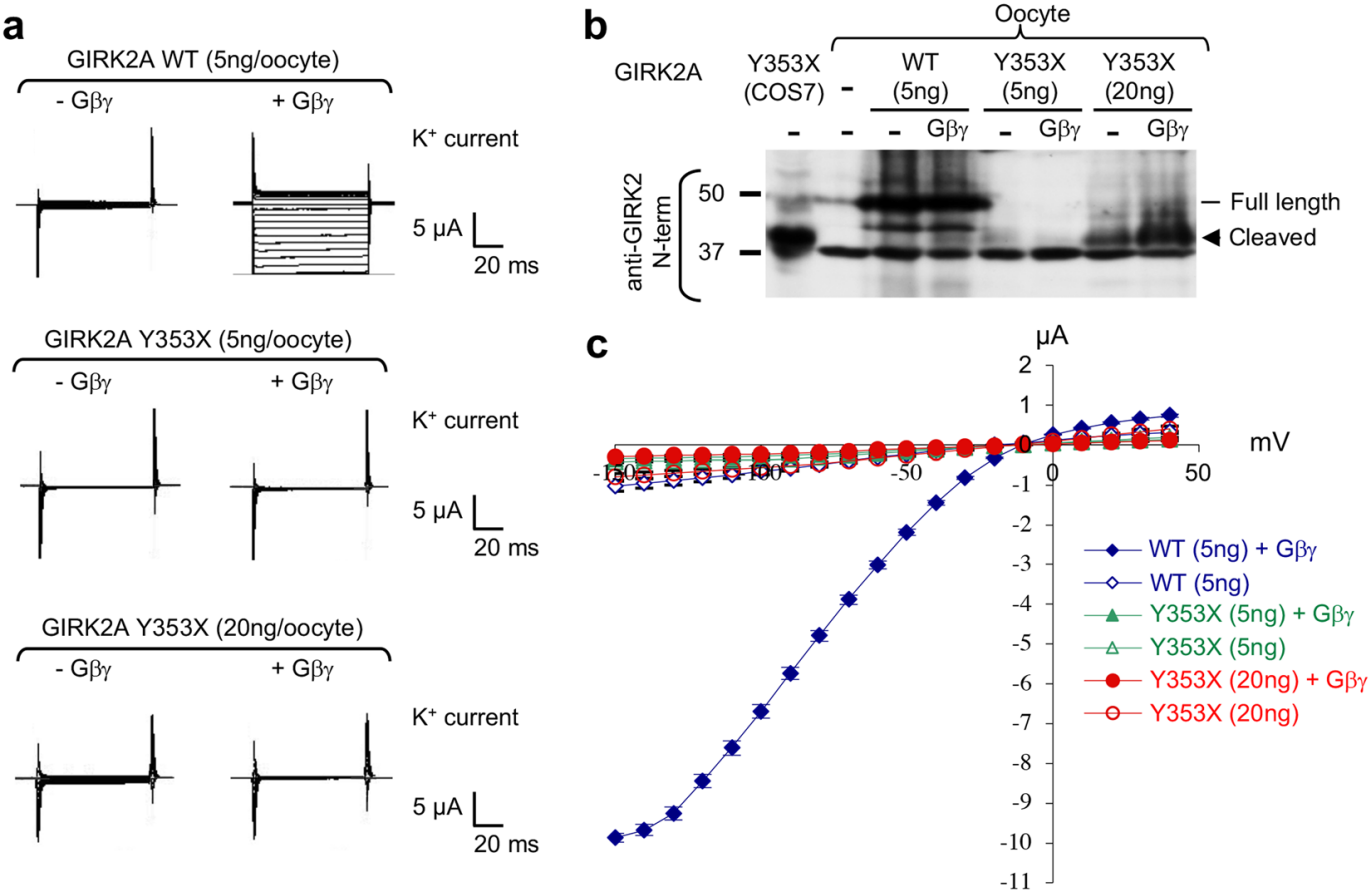
GIRK2A channels truncated at ^349^YEVD^352^ motif do not express K^+^ current. Two-electrode voltage clamp recordings of K^+^ current from *Xenopus* oocytes expressing GIRK2A WT or Y353X were performed in 90 mM KCl external bath solution upon applying the voltage steps from −150 to 40 mV in 10 mV increments. (**a**) Representative current traces from oocytes expressing GIRK2A WT and Y353X bathed in 90 mM KCl. Truncated GIRK2A-Y353X channels failed to carry K^+^ current in the presence or absence of Gβ_1_γ_2_. (**b**) Immunoblotting with anti-GIRK2 N-terminal antibodies on the lysate from COS7 cells expressing GIRK2A-Y353X and oocytes injected with cRNA of GIRK2A WT (5 ng) or Y353X (5 ng, 20 ng) with or without cRNA of Gβ_1_ and Gγ_2_ (2 ng each). The cropped gray-scale blots are displayed. Full-length blots are included in the Supplementary Fig. S6. (**c**) Representative averaged current–voltage (*I–V*) relations obtained from *Xenopus* oocytes expressing GIRK2A WT (n = 5), GIRK2A WT + Gβ_1_γ_2_ (n = 8), GIRK2A-Y353X (n = 8), GIRK2A-Y353X + Gβ_1_γ_2_ (n = 10), 20 ng GIRK2A-Y353X (n = 4), 20 ng GIRK2A-Y353X + Gβ_1_γ_2_ (n = 5). K^+^ current in oocytes expressing GIRK2A WT + Gβ_1_γ_2_ were significantly larger than oocytes expressing GIRK2A WT alone or GIRK2A-Y353X +/− Gβ_1_γ_2_ at all voltage steps except −20 to +20 mV. Data points in the *I–V* curves represent the mean ± SEM.

To test if truncated GIRK2A-Y353X channels are functional, two-electrode voltage clamp recording was performed to examine macroscopic K^+^ currents of wild type GIRK2A or truncated GIRK2A-Y353X homotetrameric channels from *Xenopus* oocytes coexpressing Gβ_1_γ_2_ subunits (Fig. 7). Wild type GIRK2A channels displayed very low level of basal K^+^ currents in the absence of Gβ_1_γ_2_, but produced significantly larger K^+^ currents upon coexpression of Gβ_1_γ_2_ (Fig. 7a,c), consistent with previous studies reporting Gβγ-dependent activation of GIRK channels^35,39^. In contrast, GIRK2A-Y353X channels produced negligible K^+^ currents whether Gβ_1_γ_2_ proteins were coexpressed or not (Fig. 7a,c). Because this lack of K^+^ currents (Fig. 7a,c) could be due to very low protein expression of GIRK2A-Y353X compared to wild type GIRK2A (Fig. 7b), we repeated the recording in the oocytes injected with 20 ng of GIRK2A-Y353X cRNA. Although these oocytes expressed increased level of GIRK2A-Y353X especially in the presence of Gβ_1_γ_2_ coexpression compared to the oocytes injected with 5 ng of cRNA (Fig. 7b), they failed to produce K^+^ currents in the presence or absence of Gβ_1_γ_2_ (Fig. 7a,c). None of the tested oocytes produced Na^+^ currents, indicating that K^+^ selectivity is intact in wild type and truncated GIRK2A channels.

### Kainate-induced status epilepticus induces C-terminal cleavage of GIRK channels in the hippocampus

**Figure 8:**
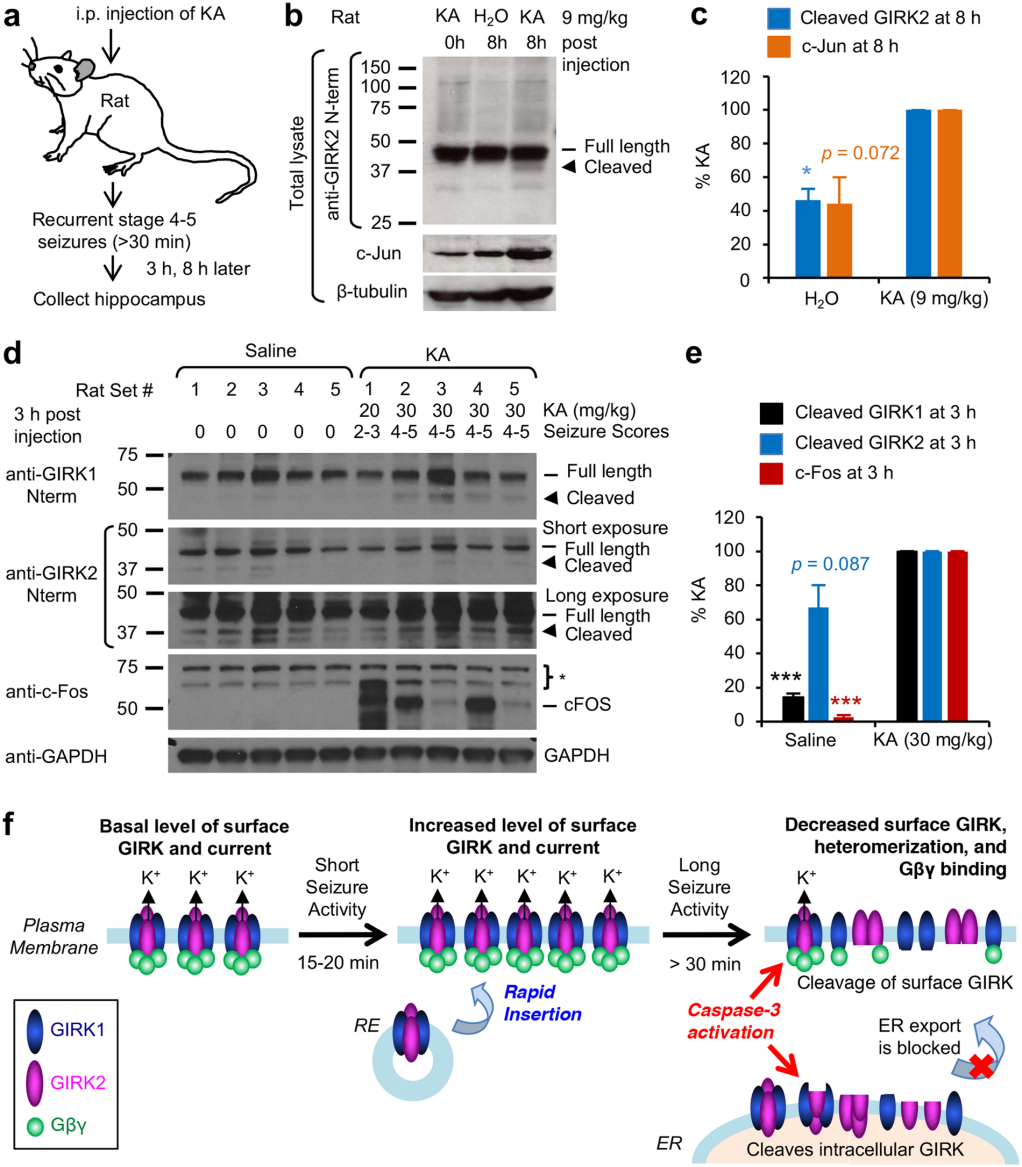
Kainate-induced status epilepticus in rats induces C-terminal cleavage of GIRK1 and GIRK2 in the hippocampus. (**a**) Schematic workflow of an experiment from kainate (KA)-mediated induction of status epilepticus in rats to collection of their hippocampi. (**b**,**c**) Immunoblot analysis of total hippocampal lysates from Sprague Dawley rats injected with kainate (9 mg/kg) and vehicle control (H_2_O) with antibodies against GIRK2 N-terminus, c-Jun (marker for seizure induction), and β-tubulin (loading control). Kainate-injected rats developed stage 4–5 seizures and status epilepticus (n = 3) whereas H_2_O-injected rats did not display seizures (n = 3). Representative immunoblots (**b**) and quantification (**c**) showed that status epilepticus induces C-terminal cleavage of GIRK2 in the rat hippocampi at 8 h post injection with 9 mg/kg kainate. (**d**,**e**) Immunoblot analysis of total hippocampal lysate from CD001 rats injected with kainate (20 and 30 mg/kg) or vehicle control (saline) with antibodies against GIRK1 and GIRK2 N-terminus, c-Fos (marker for seizure induction), and GAPDH (loading control). In one pair of CD001 rats (set #1), the rat injected with 20 mg/kg kainate developed a short duration of stage 2–3 seizures. In 4 pairs of rats (set #2–5), rats injected with 30 mg/kg kainate developed stage 4–5 seizures and status epilepticus. Saline-injected rats did not display seizures. Representative immunoblots (**d**) and quantification (**e**) showed that status epilepticus induced C-terminal cleavage of GIRK1 in the rat hippocampus within 3 h post injection with 30 mg/kg kainate (n = 4). The cropped gray-scale blots are displayed. Full-length blots are included in the Supplementary Fig. S7. Data shown represent the mean ± SEM. *p < 0.05, ***p < 0.005. (**f**) A working model for the regulation of GIRK channels by seizure activity. Induction of seizure activity increases surface expression and current of GIRK1/GIRK2 channels within 15–20 min by promoting their recycling possibly as a homeostatic defense to heightened excitability^18,19^. In contrast, prolonged seizure activity (>30 min) leads to caspase-3 dependent cleavage of GIRK1/GIRK2 channels in the ER and plasma membrane, which may likely decrease their surface and current expression, heteromerization, and/or interaction with Gβγ.

To test if prolonged epileptic seizures *in vivo* (i.e. status epilepticus) lead to the C-terminal cleavage of GIRK channels, we used a well-established rodent model of TLE in which status epilepticus was induced by intraperitoneal (i.p.) injection of kainate (Fig. 8a), a potent agonist for ionotropic glutamate receptors^4,40^. We chose the kainate model of TLE due to hippocampus-restricted injuries and histopathological correlates of hippocampal sclerosis^4,40^ associated with increased level and activation of caspase-3^41,42^. Furthermore, kainate-induced seizures have been shown to induce expression of an immediate early gene c-Fos which is involved in dampening excitability and promoting survival of hippocampal neurons^43^, as well as another immediate early gene c-Jun which is a primary substrate of c-Jun N-terminal kinases (JNKs) important for excitotoxic neuronal apoptosis in the hippocampus following status epilepticus^44^.

Injection of kainate (9 mg/kg) but not vehicle control (H_2_O) induced behavioral hyperactivity within 20 min and recurrent stage 4–5 seizures (Racine scale) and status epilepticus within 1 hour (h) in Sprague Dawley rats. Kainate-induced status epilepticus significantly increased the level of C-terminally cleaved GIRK2 subunits in rat hippocampi at 8 h post injection (Fig. 8b,c). There was an increasing trend in c-Jun expression at 8 h post kainate injection, but this trend did not reach statistical significance (Fig. 8b,c). In separate experiments, CD001 rat hippocampal lysate was prepared at 3 h post injection with vehicle control (saline) or kainate (20 and 30 mg/kg) (Fig. 8d,e). There was a low level of cleaved GIRK1 and GIRK2 proteins in the hippocampi of saline-treated CD001 rats (Fig. 8d). Injection of 20 mg/kg kainate caused stage 2–3 seizures which did not last after 1 h post injection, and did not further induce C-terminal cleavage of GIRK1 and GIRK2 at 3 h post injection (Fig. 8d). However, injection of 30 mg/kg kainate induced recurrent stage 4–5 seizures in CD001 rats which significantly increased the levels of c-Fos and C-terminally cleaved GIRK1 proteins but not the levels of C-terminally cleaved GIRK2 proteins within 3 h post injection (Fig. 8d,e). Similar to rats, C57BL/6 J mice injected with 15 mg/kg and 30 mg/kg kainate displayed recurrent stage 3–4 seizures and 4–5 seizures respectively, and displayed enhanced c-Fos and c-Jun expression and increased C-terminal cleavage of GIRK2 (Supplementary Fig. S8).

## Discussion

The intracellular N- and C-terminal tails of GIRK channel subunits provide multi-functional regions that form the cytoplasmic pore of the channels and the interacting domains for Gβγ and other signaling proteins that regulate their function and trafficking^6,45^. Here, we demonstrate a novel mode of GIRK channel regulation: caspase-3 cleavage of neuronal GIRK channel subunits GIRK1 and GIRK2 which occurs during prolonged seizure activity (Figs 1–2). Our surface biotinylation experiment showed that prolonged seizure activity induced by APV withdrawal resulted in caspase-3 dependent C-terminal cleavage of endogenous GIRK1 and GIRK2 subunits at the plasma membrane (Figs 1 and 2). Furthermore, such cleavage of GIRK subunits was also observed in total neuronal lysate which contained both surface and intracellular GIRK subunits (Figs 1 and 2). Since our previous report has shown that 7% total GIRK1 proteins and 19% total GIRK2 proteins were on the cell membrane of cultured hippocampal neurons^18^, cleavage of almost 50% total GIRK1 and GIRK2 proteins (Fig. 2) suggests that intracellular GIRK1 and GIRK2 were also cleaved by caspase-3.

To date, our studies are the first to identify caspase-3 cleavage sites in GIRK subunits (Fig. 3). The “YEVD” caspase-3 cleavage motifs and the surrounding amino acid sequences are highly conserved in the intracellular C-terminal tails of GIRK2-4 (Fig. 4) and located upstream of specific motifs that control their surface expression, intracellular trafficking, and protein-protein interaction^18,36–38^. Indeed, GIRK2A-Y353X, which mimics GIRK2A cleaved by caspase-3, failed to express on the plasma membrane of COS7 cells due to the loss of its ER export motif ^396^ELETEEE^403^ (Fig. 6). Although we cannot completely exclude the possibility that caspase cleavage at YEVD motif may directly destroy the channel activity, impairment in surface expression most likely underlies the inability of truncated GIRK2A-Y353X channels to produce current in oocytes (Fig. 7, 8f). Similarly, the loss of forward trafficking motif in GIRK4 by caspase-3 cleavage of intracellular GIRK4 (Fig. 4b) is expected to reduce GIRK4 surface expression. Since GIRK3 lacks an ER export motif and therefore requires GIRK2 for its surface expression^8,36^, caspase-3 cleavage of GIRK2 and GIRK3 subunits at the ER is expected to block the ER export of GIRK2/GIRK3 channels.

Interestingly, we observed a significantly lower expression of truncated GIRK2A-Y353X than wild-type GIRK2A (Figs 5a, 6b and 7b), suggesting the degradation of cleaved GIRK2 proteins retained in the ER. Furthermore, a marked decrease in GIRK1 surface expression was also observed in cells expressing GIRK2A-Y353X (Fig. 6) which were unable to interact with GIRK1 (Fig. 5), consistent with the report that surface expression of GIRK1 requires its interaction with GIRK2^8,36^. Reduced total expression of GIRK1 (Fig. 5) and the ladder of cleaved GIRK1 proteins in the immunoblots of cultured hippocampal neurons (Figs 1 and 2) points to the possibility of ubiquitination-mediated degradation of truncated GIRK1. These observations suggest the instability of cleaved channel portions of GIRK1 and GIRK2, although the fate of much shorter C-terminal fragments of GIRK1 and GIRK2 remains unclear.

Importantly, caspase-3 cleavage of GIRK2-4 at “YEVD” motif removes the secondary βM and βN sheet structural elements from the channel contact surface for Gβγ proteins (Fig. 4), which mediate membrane-delimited activation of GIRK channels^45^. Since truncated GIRK2A-Y353X coimmunoprecipiated with Gβγ significantly less than the wild type GIRK2A (Fig. 5), caspase-3 mediated cleavage of GIRK2 is predicted to decrease Gβγ binding to GIRK1/GIRK2 channels at the plasma membrane that constitute most neuronal GIRK channels^7^, as well as GIRK2/GIRK3 or GIRK4 channels that are expressed in a subset of neurons^8–10^. Caspase-3 cleavage site in GIRK1 ^387^ECLD^390^ (Fig. 3) is located far distal to the Gβγ contact surface of GIRK1 (Fig. 4), and thus cleavage would have minimal effect on Gβγ binding to GIRK1. However, inability of truncated GIRK2A-Y353X to interact with GIRK1 (Fig. 5) suggests that caspase-3 cleavage of GIRK2 would cause disassembly of GIRK1/GIRK2 channels, further contributing to their dysfunction (Fig. 8f).

There is emerging evidence for a macromolecular complex on the plasma membrane consisting of GIRK channels, GPCRs, G-protein subunits, and signaling proteins such as regulators of G protein signaling^11,46–48^ that support membrane-delimited opening of GIRK channels upon activation of GPCRs^45^. Furthermore, Gβγ binding is shown to strengthen the interaction between GIRK channels and phosphatidylinositol-4,5-bisphosphate (PIP_2_), an essential co-factor for channel gating^32,49^. We speculate that caspase-3 cleavage of GIRK2-4 on the plasma membrane and subsequent reduction in Gβγ and GIRK1 binding would destabilize this macromolecular complex and PIP_2_ interaction, decoupling the activation of GPCRs from the membrane-delimited gating of GIRK channels.

What are the physiologic consequences of caspase-3 mediated cleavage of GIRK subunits? GIRK channels regulate resting membrane potential and excitability in hippocampal neurons by mediating inhibitory effects of GPCRs for neurotransmitters and neuromodulators, including adenosine A1 receptors and GABA_B_ receptors^6^. Our current study has observed that surface expression of GIRK1 and GIRK2 is increased within 15–30 min of seizure activity (Figs 1 and 2). These results are consistent with our previous studies^18,19^, which report that this regulation involves enhanced recycling of GIRK channels to the plasma membrane, and is associated with increased basal GIRK current and GIRK channel activation induced by adenosine A1 receptors but not GABA_B_ receptors^18,19^. Such initial upregulation of GIRK surface density may likely provide homeostatic defense to reduce neuronal excitability against seizure activity.

However, when this seizure activity persisted for >30 min, we discovered a novel finding that surface and total GIRK1 and GIRK2 proteins are cleaved by caspases sensitive to potent caspase-3 inhibitor DEVD-fmk with *Ki* of 0.2 nM (Fig. 2). Since DEVD-based peptide inhibitor also blocks other apoptosis executioner caspase-7 (*Ki* = 1.6 nM), as well as upstream apoptosis initiators caspase-8 (*Ki* = 0.9 nM) and caspase-10 (*Ki* = 12 nM)^50^, the identity of caspases that cleave GIRK1 and GIRK2 during prolonged seizure activity in hippocampal neurons remains unclear. Nonetheless, we demonstrated that GIRK2A-Y353X, which mimics GIRK2 cleaved at YEVD motif by caspase-3, had decreased binding to Gβγ and GIRK1 and failed to display surface and current expression (Figs 4–7). Based on our findings and the reports of increased spontaneous sporadic and lethal seizures in GIRK2 knock-out mice^16^, we propose that caspase-mediated cleavage and subsequent down-regulation of GIRK channels may decrease their basal and GPCR-activated K^+^ current and disrupt their ability to dampen excitability (Fig. 8f). Future studies are needed to determine the role of caspase-mediated cleavage of GIRK in neuronal excitability.

The possible involvement of apoptosis initiators caspase-8 and 10 that can also cleave at DEVD and XEXD^29,30^ motifs is intriguing, since earlier cleavage of GIRK1 and GIRK2 by these caspases could potentially alter neuronal physiology before cell death. Furthermore, in addition to a key role of caspase-3 in terminal apoptosis execution events^51^, recent studies have revealed apoptosis-independent roles of caspase-3 in neurons such as synaptic plasticity^52,53^ including NMDAR-dependent long-term depression of excitatory synapses^54,55^. Interestingly, we observed a low level of cleaved GIRK1 and GIRK2 subunits in cultured hippocampal neurons under APV control condition (Figs 1 and 2) and in the hippocampi of vehicle-treated control rats (Fig. 8), which could be caused by caspase-8 and 10 or apoptosis-independent caspase-3 activity. GIRK channels and GABA_B_ receptors are detected in dendritic spines that harbor the majority of excitatory synapses in hippocampal neurons^11^ where their activation provides slow inhibitory postsynaptic currents^15^. In addition to postsynaptic roles, activation of GIRK channels by GABA_B_ receptors in presynaptic terminals has been shown to inhibit neurotransmitter release^56,57^. Furthermore, both GIRK channels and A_1_ receptors reside on the dendritic spines and shafts^11,15,58,59^ where GIRK activation by adenosine attenuates the excitatory postsynaptic potentials^14^ and contributes to depotentiation of NMDAR-dependent long-term potentiation^19^. Therefore, it is tempting to speculate that caspase-mediated cleavage of GIRK1 and GIRK2 may disrupt GIRK-mediated synaptic inhibition and/or plasticity before neurons are fully committed to apoptosis.

We have also discovered that kainate-induced status epilepticus in rats leads to C-terminal cleavage of GIRK1 and GIRK2 in their hippocampi (Fig. 8) where GIRK1-3 subunits are expressed with overlapping distribution patterns^60^. Studies in human TLE and kainate rodent models of TLE indicate that excessive glutamate release during status epilepticus causes hippocampal neuronal death by a combination of necrosis and apoptosis depending on the seizure intensity and the cellular energy levels^3,5^. In addition, activation of caspase-2, 3, 6, 7, and 8 has been observed in the hippocampi of human TLE patients and rodent models of TLE where activity of caspase-3 and 7 is highly correlated with hippocampal sclerosis^42^. In cultured hippocampal neurons, apoptosis induced by prolonged high-frequency epileptiform discharges is evident by DNA fragmentation and activation of caspase-3 family proteins^20,21^, and is blocked by pharmacological inhibition of NMDARs^20^. Indeed, one important culprit for glutamate-induced neuronal death has been shown to be massive accumulation of intracellular Ca^2+^ upon overstimulation of NMDARs^61^. Though highly speculative, caspase-mediated cleavage and down regulation of neuronal GIRK channels may cause sustained depolarization and overstimulation of NMDARs, creating pathologic positive feedback mechanisms that amplify Ca^2+^ overload and sensitize the neurons to Ca^2+^-induced excitotoxic death. This drastic measure could allow the brain to eliminate the neurons that persistently produce epileptiform discharges when initial homeostatic mechanisms fail to dampen their excitability, and may in part underlie sclerosis at or close to the seizure foci in the hippocampi of human TLE patients^3^. Testing this hypothesis warrants future studies. Lastly, GIRK channels may likely represent one of many caspase-3 targets, and therefore future research shall include a more comprehensive identification of other caspase-3 substrates involved in seizure-induced hippocampal neuronal injury.

## Methods

### Experimental animals

All procedures involving animals were reviewed and approved by the Institutional Animal Care and Use Committee at the University of Illinois Urbana-Champaign and the University of California San Francisco (UCSF) in accordance with the guidelines of the U.S National Institutes of Health (NIH).

### Induction of seizure activity in hippocampal neuronal culture

Primary dissociated hippocampal neuronal cultures at high density (330 cells/mm^2^) were prepared from Sprague-Dawley rat embryos at E18 as described^18^. Cultured neurons (10–11 DIV) were treated for 2–3 days with DL-APV (200 μM, Tocris). To induce seizure activity, APV-treated neurons were incubated with “APV withdrawal” ACSF solution containing (in mM): 10 HEPES, 145 NaCl, 2.5 KCl, 2 CaCl_2_, 10 Dextrose, 0.1 glycine, 0.1 picrotoxin, and 0.005 strychnine (Sigma, Tocris) as described^18^. For control, APV-treated neurons were incubated with “APV control” ACSF containing (in mM): 10 HEPES, 145 NaCl, 2.5 KCl, 2 CaCl_2_, 1 MgCl_2_, 10 Dextrose, 0.1 picrotoxin, 0.005 strychnine, 0.2 DL-APV (pH 7.4, 305–315 mOsm). Neuronal lysates were prepared in RIPA buffer and immunoblotted with antibodies against GIRK1 and GIRK2 N-termini (1:200)^18^ (Supplementary Fig. S2) and glyceraldehyde-3-phosphate dehydrogenase (GAPDH, 1:1,000). ImageJ software (NIH, http://rsb.info.nih.gov/ij) was used to measure background-subtracted immunoblot band intensities of full-length GIRK, cleaved GIRK, and GAPDH. The ratio of GIRK/GAPDH from APV control samples (Figs 1c,d and 2c,d) were taken as 100% and the ratio of APV withdrawal samples were normalized to the ratio of APV control samples to obtain % APV control.

### Whole cell patch clamp recordings

Current clamp recordings of spontaneous firings from hippocampal pyramidal neurons were carried out at 23–25 °C using gap free mode as described^19^ first in “APV control” ACSF for 10 min, and then in “APV control” or “APV withdrawal” ACSF for 15 min. Recording pipettes had a resistance of 3–5 MΩ when filled with internal solution containing (in mM): 130 K-Gluconate, 7 KCl, 2 NaCl, 10 HEPES, 1 MgCl_2_, 0.1 EGTA and 2 ATP-Mg, 0.3 Na-GTP (pH 7.3, 285–295 mOsm). Recordings were performed using a Multiclamp 700B amplifier, Digidata 1440 A, and the pClamp 10.6 (Molecular Devices), filtered at 2 kHz, digitized at 10 kHz, and analyzed using Clampfit 10.6 (Molecular Devices).

### Surface biotinylation

After APV control or withdrawal, surface biotinylation was performed with Sulfo-NHS-SS (or LC)-Biotin (1 mg/mL, Pierce) in ACSF on ice as described^18^. Biotinylated proteins were precipitated using 50% NeutraAvidin agarose (200 μL Pierce) and eluted in SDS sample buffer upon heating at 85 °C for 30 min. For inhibition of caspases, APV-treated neurons were preincubated for 2 h with ZVAD-fmk (100 μM, Enzyme Systems), YVAD-cmk (20 μM, Clontech), or DEVD-fmk (20 μM, Calbiochem) before APV control or withdrawal.

### DNA constructs and mutagenesis

The plasmids pcDNA3 containing GIRK1 (NM_031610.3), GIRK2A (NM_010606.2), GIRK4A (NM_017297), GIRK1 with an extracellular HA tag (HA-GIRK1), and pCMV5 containing GIRK2A with an extracellular HA tag (HA-GIRK2A) have been described^18^ (kind gifts from Dr. Lily Y. Jan, UCSF). Mutations in GIRK1 (D390E, D393E), GIRK2A (D346E, D352E, Y353X), and GIRK4 (D347E) were generated using the QuikChange II XL Site-Directed Mutagenesis Kit (Agilent), and verified by sequencing the entire cDNA construct. Mutagenesis oligonucleotides were: GIRK1-D390E (sense ^5′^CAATTCTGTGGAGTGCTTAGAGGGACTA GATGACATTAGC^3′^, antisense ^5′^GCTAATGTCATCTAGTCCCTCTAAGCACTCCACAGA ATTG^3′^), GIRK1-D393E (sense ^5′^GTGCTTAGATGGACTAGAGGACATTAGCACAAAA CTTCC^3′^, antisense ^5′^GGAAGTTTTGTGCTAATGTCCTCTAGTCCATCTAAGCA^3′^), GIRK2-D346E (sense ^5′^GTCCTAACGCTGGAAGAAGGGTTCTACGAAGTTG^3′^, antisense ^5′^CAAC TTCGTAGAACCCTTCTTCCAGCGTTAGGAC^3′^), GIRK2-D352E (sense ^5′^CGGGTTCTACGAAGTTGAGTACAACAGCTTCCATG^3′^, antisense ^5′^CATGGAAGCTGTTGTACTCAAC TTCGTAGAACCCG^3′^), GIRK2-Y353X (sense ^5′^GTTCTACGAGTTGACTAGAACAGC TTCCATGAGAC^3′^, antisense ^5′^GTCTCATGGAAGCTGTTCTAGTCAACTTCGTAGAAC^3′^), and GIRK4-D347E (sense ^5′^GGCTTCTATGAGGTGGAGTACAACACTTTCCACG^3′^, antisense ^5′^CGTGGAAAGTGTTGTACTCCACCTCATAGAAGCC^3′^). The underlined bases indicate amino acid substitution mutations in the sense and antisense sequences.

### *In vitro* cleavage reaction

The pcDNA3 plasmids containing wild type or mutant GIRK cDNA (1 μg) were incubated with the TnT coupled Reticulocyte Lysate System (50 μL, Promega) and L-[^35^ *S]-Methionine* (>1,000 Ci/mmol at 10 mCi/ml) (Amersham Biosciences) for 90 min at 30 °C. This system utilizes crude reticulocyte lysate which could contain microsomes, and has been widely used to synthesize membrane proteins *in vitro* in a cell-free system^62^. Translated proteins were subjected to *in vitro* cleavage reaction at 37 °C for 2 h with 0.2 μg caspase-1 (Calbiochem) or caspase-3 (BD Biosciences) in a buffer containing (in mM): 20 PIPES, 100 NaCl, 10 DTT, 1 EDTA, 0.1% CHAPS, and 10% sucrose. Products were separated by electrophoresis and visualized by exposure to autoradiographic BioMax MS films (Kodak).

### Visual molecular dynamics

Crystal structures of GIRK2 alone or GIRK2 complexed with Gβγ (protein data bank ID code 4kfm)^34^ were rendered with Visual Molecular Dynamics software^63^ to create molecular surface representation of GIRK2 in blue and Gβγ in green.

### Coimmunoprecipitation

For coimmunoprecipitation (coIP) of GIRK2A and Gβγ, HEK293T cells were transfected using FuGENE6 transfection reagent (Promega) with pcDNA3.1-YFP-G-protein Gβ_1_ (0.4 μg), pcDNA3.1-YFP-G-protein-Gγ_2_ (0.4 μg) (kind gifts from Narasimhan Gautam, Addgene plasmids #36397, #36102) and pcDNA3-GIRK2A wild-type (0.8 or 1 μg) or Y353X (0.8 or 2 μg). Immunoprecipitation (IP) was performed at 24 h post transfection as described^64^ with Protein A/G agarose beads (50 μL) and mouse anti-GFP antibodies (5 μg, Cell Signaling). After washing, the beads were incubated with SDS sample buffer at 90 °C for 5 min. The lysates and IP eluates were immunoblotted with antibodies for GFP, GIRK2 N-term and GAPDH (1:500–1:1,000). For coIP of GIRK2A and HA-GIRK1, HEK293T cells were transfected with pcDNA3.1-HA-GIRK1 (1 μg), and pcDNA3-GIRK2A wild type (1 μg) or Y353X (2 μg). IP was performed with rabbit anti-GIRK2 N-terminal antibodies (5 μg, Cell Signaling) and analyzed by immunoblotting with anti-HA, anti-GIRK2 N-term, anti-GAPDH antibodies (1:500–1:1,000) (Cell Signaling). To quantify coIP, background-subtracted IP band intensity ratios of GIRK2A/YFP- Gβ_1_ or HA-GIRK1/GIRK2A from GIRK2A Y353X samples were normalized to the ratios from GIRK2A WT samples to obtain % GIRK2A WT (Fig. 5b) or % HA-GIRK1 + GIRK2A WT (Fig. 5c).

### Immunocytochemistry

At 24 h post transfection with pCMV5-HA-GIRK2A (for GIRK2A channels), or pcDNA3-HA-GIRK1 with pcDNA3-GIRK2A (for HA-GIRK1/GIRK2A channels), COS7 cells were fixed with 4% formaldehyde/4% sucrose in PBS for 8 min and incubated with anti-HA antibodies (1:300, Cell Signaling) followed by incubation with Alexa488- or Alexa594-conjugated secondary antibodies, respectively (1:200, Invitrogen). In a separate experiment, biotin-conjugated secondary antibodies were used followed by incubation with Alexa488- or Alexa594-conjugated streptavidin, respectively. After fixation and permeabilization with 0.2% Triton X-100 in PBS for 10 min, total HA-GIRK2A, HA-GIRK1, or GIRK2A proteins were labeled with anti-HA or anti-GIRK2 antibodies (1:300, Cell Signaling), followed by Alexa594-, Alexa488-, or Alexa680-conjugated secondary antibodies, respectively. High-resolution gray scale images of immunostained cells were acquired using the same exposure time under a Zeiss Axiovert 200 M inverted microscope equipped with AxioCam HRm Camera and Axiovert software. Background-subtracted mean fluorescence intensities of immunostained cells were measured using ImageJ Software as described^18,64^.

### Two electrode voltage clamp recordings

After linearizing the plasmids pLin-GIRK2A, pFroggy-Gβ_1_, pFroggy-Gγ_2_^18,39^, capped cRNA were synthesized using the Amplicap T7 or Sp6 High Yield Message Maker kit (Epicentre Technologies). Stage V–VI *Xenopus laevis* oocytes were injected with cRNA (2 ng for Gβ_1_ and Gγ_2_, 5 ng for GIRK2A, 5 and 20 ng for GIRK2A-Y353X), and maintained at 16 °C in ND96 solution containing (in mM): 96 NaCl, 2 KCl, 1 MgCl_2_, 5 HEPES (pH 7.4). Macroscopic currents were recorded from oocytes at 23–25 °C using GeneClamp 500B (Axon Instruments). Electrodes filled with 3 M KCl had a resistance of 0.4–1 MΩ. To assess the resting potential and leakiness of the cell, initial and final recordings were performed in “90 mM NaCl” external bath solution containing (in mM): 90 NaCl, 2 MgCl_2_, and 10 HEPES (pH 7.4). To record K^+^ current, “90 mM NaCl” solution was changed to “90 mM KCl” bath solution containing (in mM): 90 KCl, 2 MgCl_2_, and 10 HEPES (pH 7.4) using a ValveLink 16 perfusion system (AutoMate Scientific).

### Kainate-induced status epilepticus

To elicit status epilepticus, male rodents were subjected to intraperitoneal (i.p.) injection of vehicle control (autoclaved H_2_O or saline) or kainate (abcam) as described^40^ at 9 mg/kg for Sprague-Dawley rats weighing 200–250 g (Fig. 8b,c), 20 and 30 mg/kg for CD001 rats weighing 110–160 g (Fig. 8d,e), 15 and 30 mg/kg for C57BL/6 J mice weighing 20 g (Supplementary Fig. S8). Rodents were returned to their home cage and video monitored. At 3 h and 8 h post injection, 2 hippocampi per rat was homogenized in modified RIPA buffer (1 mL) containing 1% SDS and protease inhibitors, sonicated briefly, and centrifuged at 14,000 rpm. Two hippocampi per mouse was homogenized as described^65^ to isolate crude homogenate (S1), soluble (S2) and membrane (P2) protein fractions. Hippocampal lysates were immunoblotted with antibodies for GIRK1 and GIRK2 N termini (1:200, Supplementary Fig. S2), c-Fos, c-Jun, anti-β-tubulin, or GAPDH (1:500–1:1,000, all Cell Signaling). Several hippocampal lysates from vehicle-treated rats did not show any immunoblot bands for cleaved GIRK (Fig. 8b–e). Therefore, background-subtracted immunoblot band intensity ratios of cleaved GIRK/loading controls (β-tubulin or GAPDH) from kainate-injected rats were taken as 100%. The ratio from vehicle-injected rats were then normalized to the ratio from kainate-injected rats to obtain % KA.

### Statistical Analyses

Using Origin 9.1 (Origin Lab), the Student’s *t* test and one-way ANOVA with Fisher’s multiple comparison test were performed to identify the statistically significant difference with a priori value (*p*) < 0.05 between 2 groups and for >3 groups, respectively.

### Data Availability

The datasets generated during and/or analyzed during the current study are available from the corresponding author on reasonable request.

## Electronic supplementary material

Supplementary Information
