# Supplementary material for: Prolonged seizure activity causes caspase dependent cleavage and dysfunction of G-protein activated inwardly rectifying potassium channels
Source: Sci Rep. 2017 Sep 26;7:12313. doi: 10.1038/s41598-017-12508-y (PMC5615076; doi:10.1038/s41598-017-12508-y)

Prolonged seizure activity causes caspase dependent cleavage and dysfunction of G-protein activated inwardly rectifying potassium channels.

Brian C. Baculis[1,2,#], Amanda C. Weiss[1,#], Weilun Pang[1,#], Han Gil Jeong[1], Jun Hee Lee[1], Dai-Chi Liu[1,2], Nien-Pei Tsai[1,2], Hee Jung Chung[1,2],

[1]Department of Molecular and Integrative Physiology, [2]Neuroscience Program, and University of Illinois at Urbana-Champaign, Urbana, Illinois 61801, USA.

SUPPLEMENTARY METHODS:

Multi-electrode array (MEA) recordings. Primary dissociated rat hippocampal neurons (1.5 x10^5 cells) were plated onto poly D-lysine (0.1 mg/mL, Sigma)-coated MEA electrode area containing 64 electrodes per plate (Axion Biosystem). Neurons were fed twice a week by removing half of the medium and adding fresh medium. At 11 days in vitro (DIV), neurons were treated for 2 days with DL-APV (200 μM). At DIV 13, field potentials at each MEA electrode relative to a ground electrode were recorded with a sampling rate of 25 kHz using Axion Muse 64 channel system inside a MEA recording incubator (5% CO_2, 37°C) as described[1]. To start, DL-APV (200 μM), PTX (100 μM), and strychnine (5 μM) were added to the original medium in the MEA plate in bio-safety cabinet. The plate was immediately placed in the MEA recording incubator for the "APV control" recording for 30 min. The MEA plate was removed from the recording chamber, and all medium was replaced with the saved original medium from the last feeding, which were supplied with glycine (100 μM), PTX (100 μM), and strychnine (5 μM). The MEA plate was returned to the recording incubator for "APV withdrawal " recording for additional 90 min. Data was analyzed using Axion Integrated studio (AxIS) as described[1]. Raster plots were generated using NeuralMetricTool software.

REFERENCE:

Jewett, K.A. *et al*. Feedback modulation of neural network synchrony and seizure susceptibility by Mdm2-p53-Nedd4-2 signaling. *Mol Brain*. 9:32. doi: 10.1186/s13041-016-0214-6 (2016).

SUPPLEMENTARY INFORMATION:

Supplementary Figure S1. Synaptic NMDAR activation upon APV withdrawal increased burst firing of action potentials that lasted up to 90 min. (A) Flow chart of multi-electrode array (MEA) field potential recordings under APV control (ctl) for 30 min and APV withdrawal (wd) for 90 min from the same MEA dish containing cultured hippocampal neurons (DIV 13) pretreated with DL-APV (200 μM) for 2 days. (B, C) Representative traces of recordings from all 64 electrodes in one MEA plate. (B) Spikes per electrode from all 64 electrodes (top) and Raster plots showing bursting activities from 64 electrodes (bottom). (C) Sum of spikes from 64 electrodes. (D) AxIS data analyses of MEA recordings (n = 64 electrodes). Adaptive threshold crossing method was used for single spike detection settings to determine spike activity from noise. Inter-spike interval (ISI) threshold algorithm was used for burst detection settings with maximum ISI of 100 ms and minimum 5 spikes per single burst. Because treatment and removal of medium as well as physical movement of the MEA plates before and after recording is shown to perturb network activity of neurons on the plate, the first 15 min of recording data in APV control and APV withdrawal were omitted from the statistical analyses. Data shown represent the mean ± SEM. *$p < 0.05$, ***$p < 0.005$.

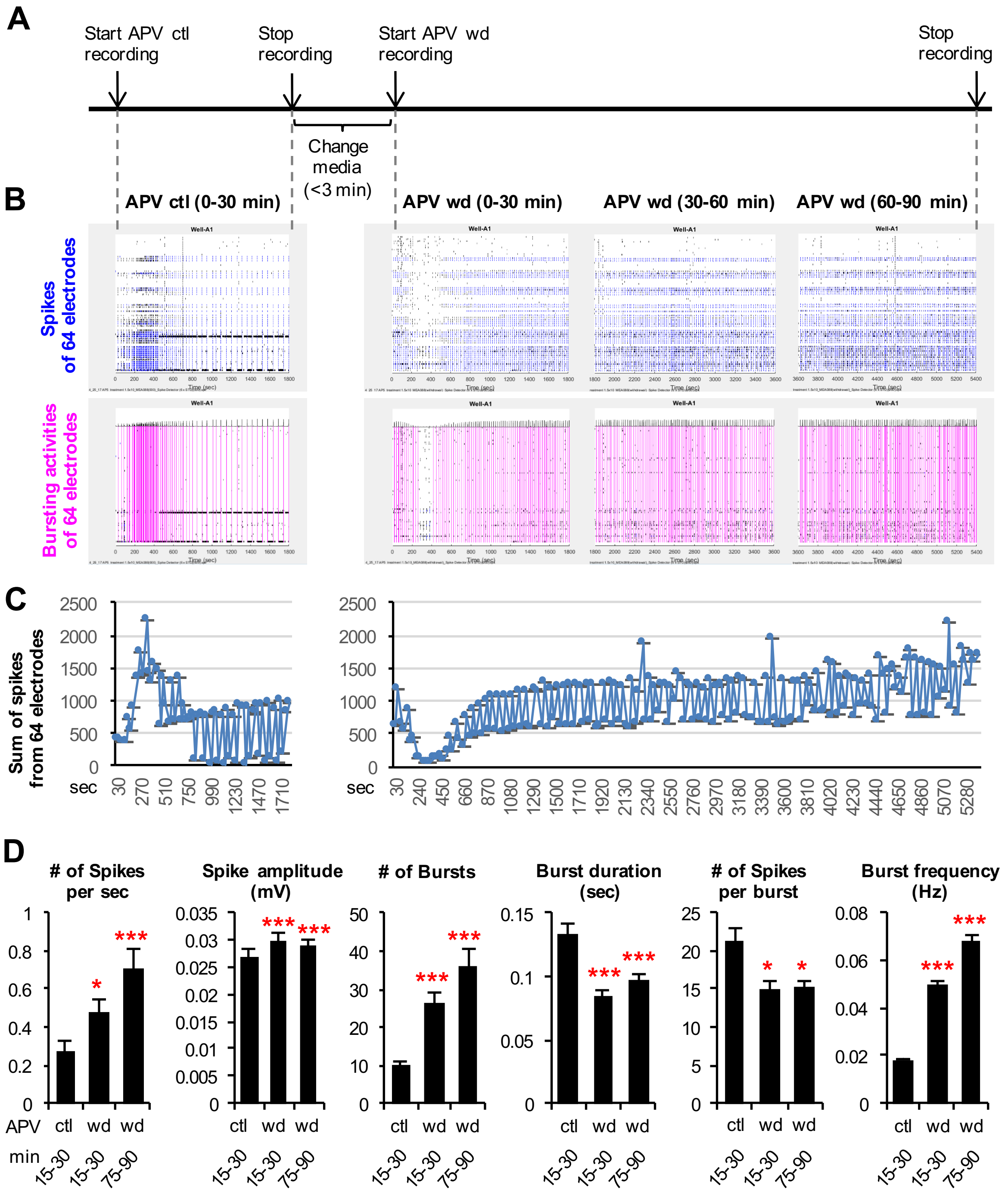
A
Start APV ctl recording
Stop recording
Start APV wd recording
Stop recording
Change media (<3 min)
B
APV ctl (0-30 min)
APV wd (0-30 min)
APV wd (30-60 min)
APV wd (60-90 min)
Spikes of 64 electrodes
Bursting activities of 64 electrodes
C
Sum of spikes from 64 electrodes
sec
D
# of Spikes per sec
Spike amplitude (mV)
# of Bursts
Burst duration (sec)
# of Spikes per burst
Burst frequency (Hz)
APV ctl wd wd
min 15-30 15-30 75-90

Supplementary Figure S2. Specificity of anti-GIRK1 and GIRK2 N-terminal antibodies used in Figures 1, 2, 7, and 8. Rabbit polyclonal anti-GIRK1 and GIRK2 N-terminal antibodies were affinity-purified from sera by chromatography. (A) Characterization of anti-GIRK1 N-terminal antibodies in HEK293T cells that were untransfected (none) or transfected with HA-GIRK1. * points at non-specific bands. (B, C) Characterization of anti-GIRK2 N-terminal antibodies in HEK293T cells that were untransfected (none) or transfected with HA-GIRK1 or HA-GIRK2A (B) or HA-GIRK1 and GIRK2A wild type (WT) or GIRK2A-Y353X in which Y353 residue right after ^{349}YEVD^{352} motif was mutated to a stop codon. Expression of HA-GIRK1 and HA-GIRK2A were also examined by anti-HA antibodies (A, B).

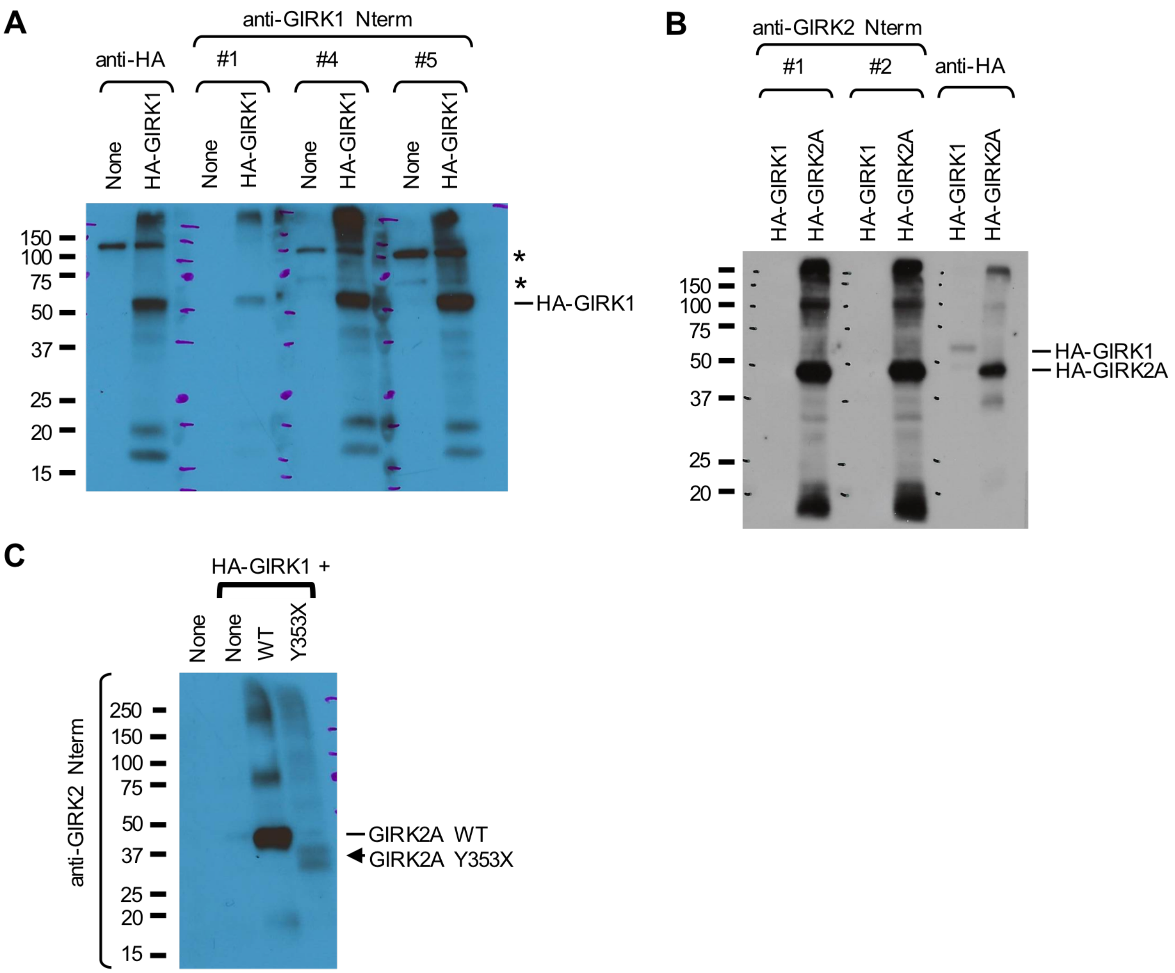

Supplementary Figure S3. Full-length immunoblots for cropped blots shown in Figure 1b-d. (A) Surface biotinylation of cultured hippocampal neurons after APV control (ctl) or APV withdrawal (wd) was immunobloted with anti-GIRK1 and GIRK2 N-terminal antibodies. (B, C) Total lysate of cultured hippocampal neurons after APV ctl or APV wd was immunoblotted with antibodies for GIRK2 N-terminus (B) and GIRK1 N-terminus (C).

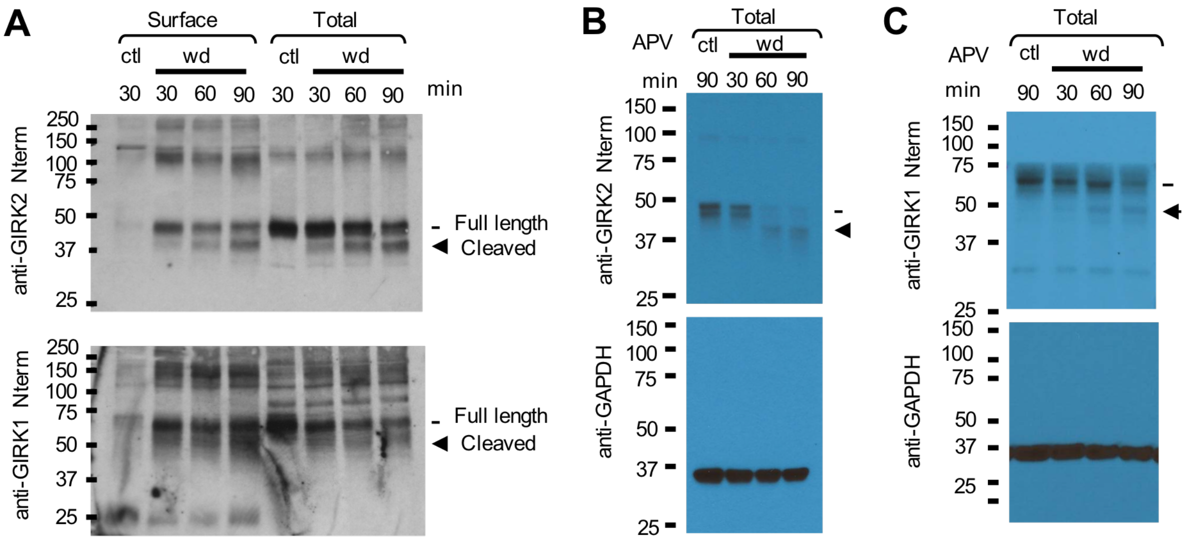

Supplementary Figure S4. Full-length immunoblots for cropped immunoblots shown in Figure 2. (A, B) Surface biotinylation was performed after APV control (ctl) or APV withdrawal (wd) in cultured hippocampal neurons pretreated for 2 h with vehicle control (VC, 0.1% DMSO), pan caspase inhibitor ZVAD-Fmk (I, 100 μM) (A), or caspase-1 inhibitor YVAD-cmk (I-1, 20 μM), or caspase-3 inhibitor DEVD-fmk (I-3, 20 μM) (B). (C, D) Immunoblot analyses were performed after APV ctl or APV wd for 120 min in cultured hippocampal neurons pretreated for 2 h with VC, ZVAD-Fmk (I), DEVD-fmk (I-3), or YVAD-cmk (I-1). * points at non-specific bands.

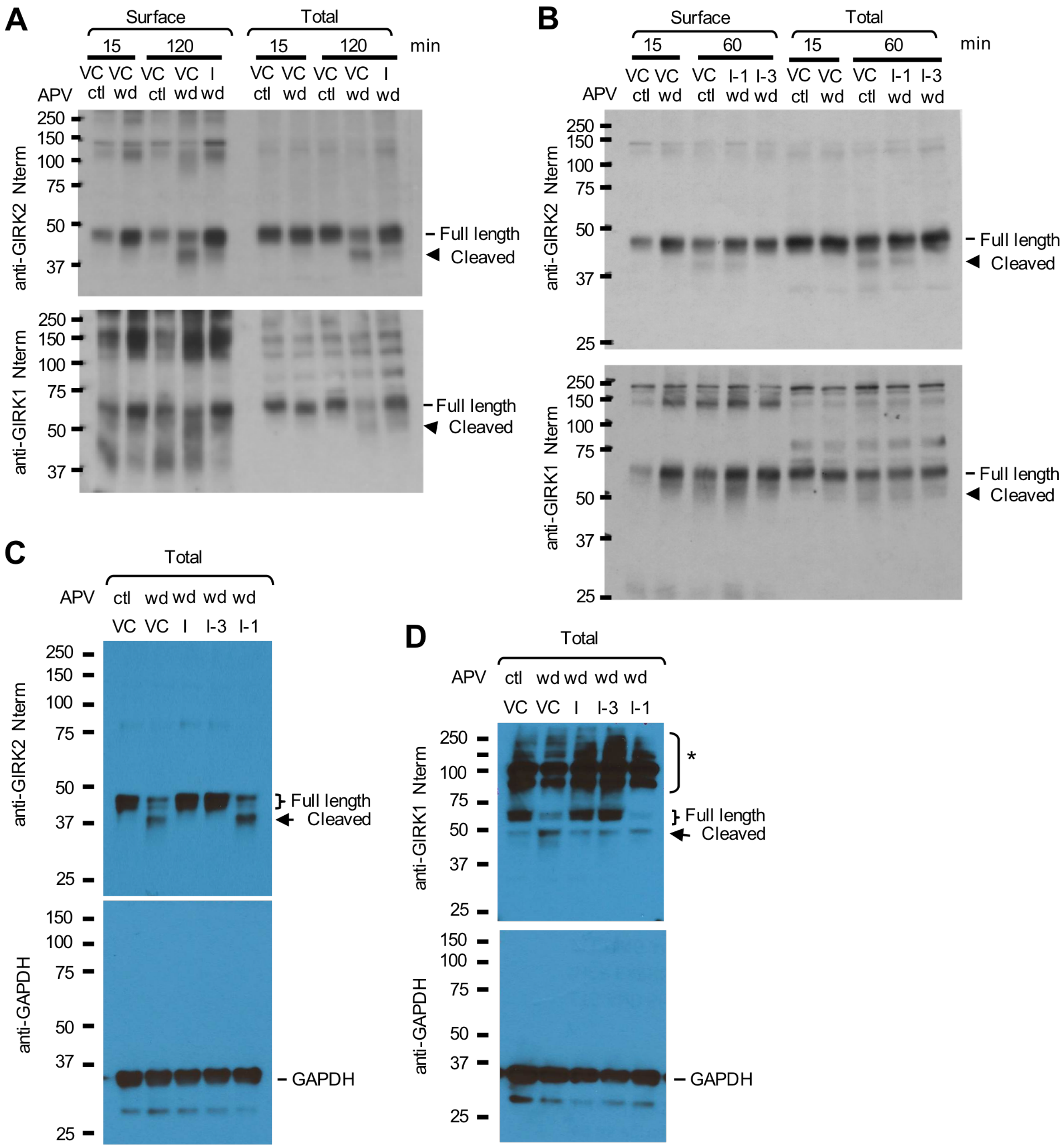

Supplementary Figure S5. Full-length immunoblots for cropped immunoblots shown in Figure 5. (A) Immunoblot analyses of HEK293T cells expressing GIRK2A wild type (WT) or Y353X with or without HA-GIRK1. The cropped gray-scale blots are displayed in Figure 5A. GAPDH served as a loading control. * points at non-specific bands. (B, C) Immunoprecipitation of YFP-Gβ_1 and YFP-Gγ_2 (B) or HA-GIRK1 (C) from the HEK293T lysates co-expressing

GIRK2A WT or Y353X. * points at IgG bands. All antibodies used for immunoprecipitation and immunoblotting were from Cell Signaling.

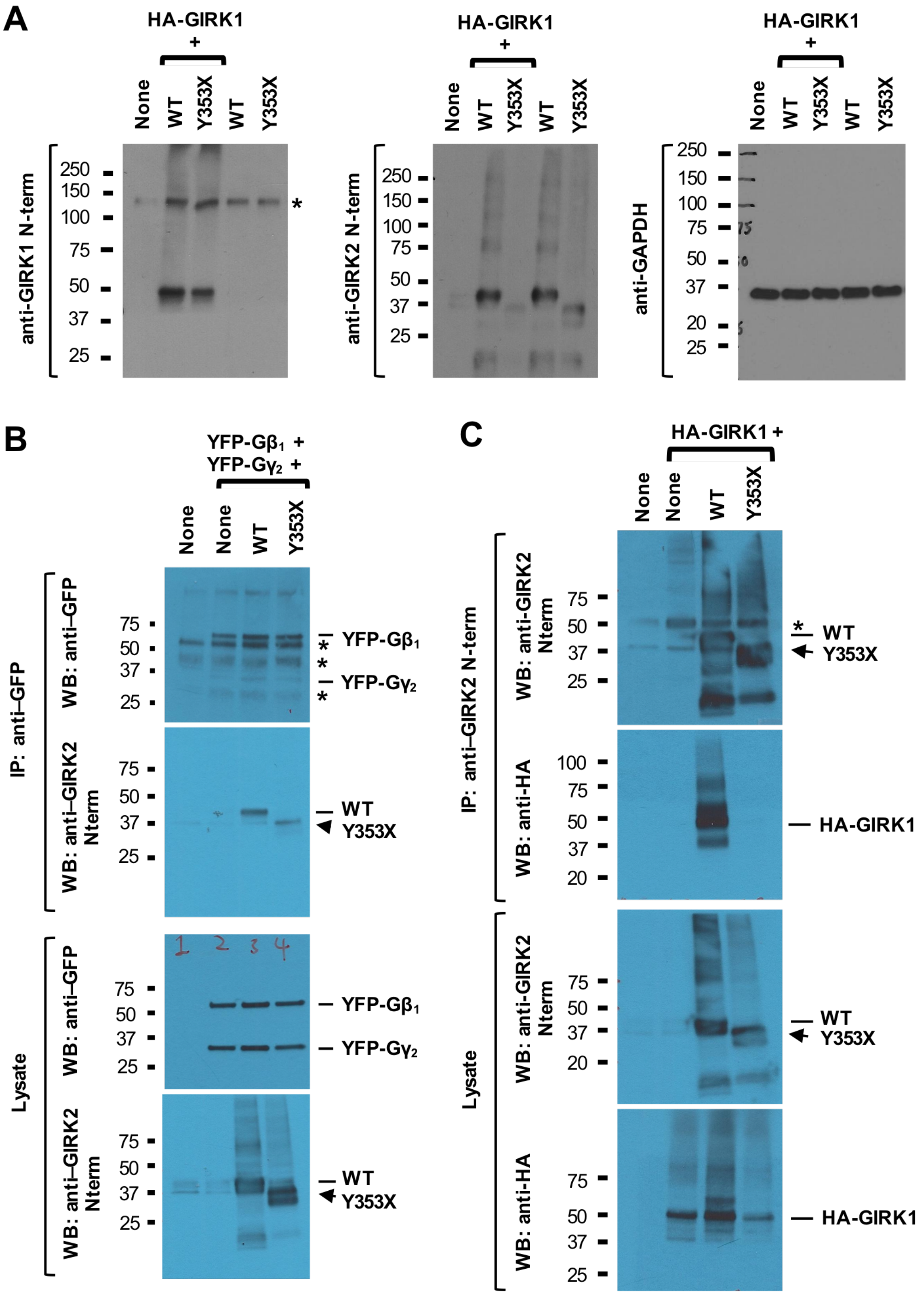

Supplementary Figure S6. Full-length immunoblot for a cropped blot in Figure 7b. Immunoblotting of COS7 cells expressing GIRK2A-Y353X and the occytes injected with GIRK2A wild type (WT) cRNA (5 ng) or Y353X cRNA (5 ng, 20 ng) with or without $G\beta_1$ (2 ng) and $G\gamma_2$ (2 ng). * points at non-specific bands.

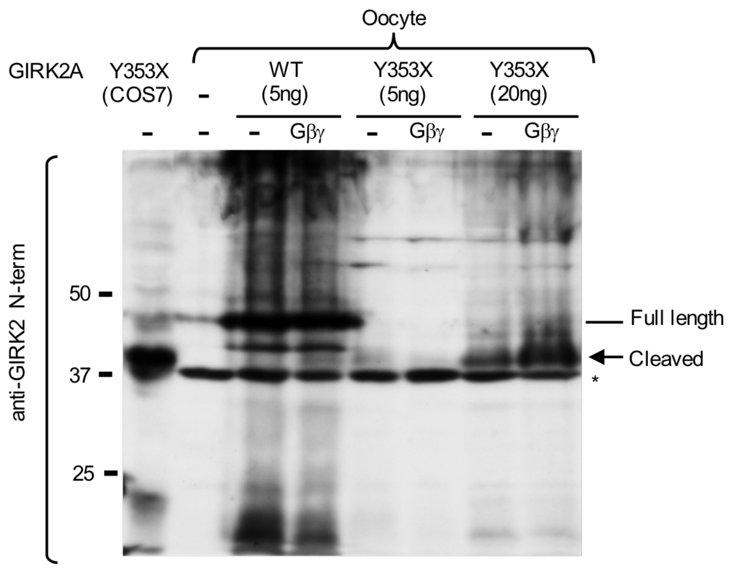

Supplementary Figure S7. Full-length immunoblots for cropped immunoblots shown in Figure 8d. Immunoblotting of hippocampal homogenate from rats at 3 h after i.p. injection withj kainate (15 and 30 mg/kg) or vehicle control (saline) using antibodies against GIRK1 and GIRK2 N-termini (described in Supplementary Fig. S2), c-FOS, and GAPDH (all from Cell Signaling). * points at non-specific bands.

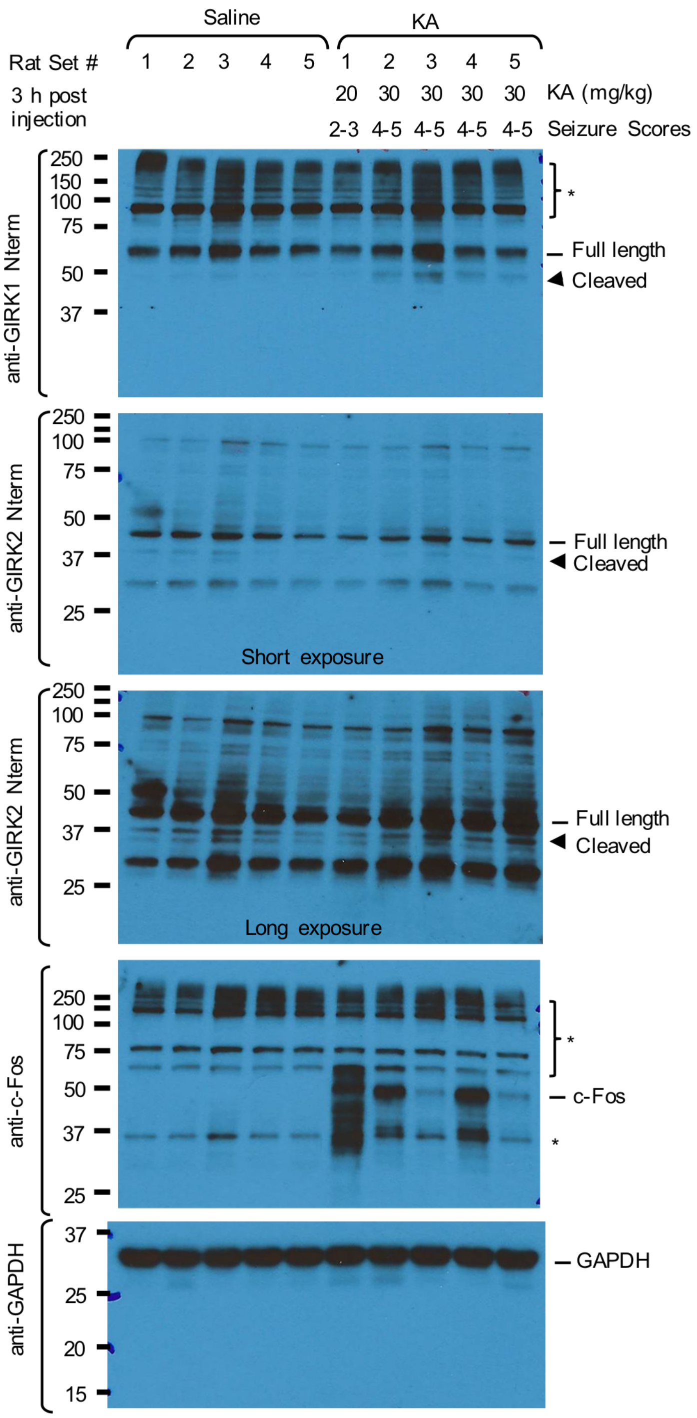
Saline
KA
Rat Set #
1 2 3 4 5 1 2 3 4 5
3 h post injection
20 30 30 30 30 KA (mg/kg)
2-3 4-5 4-5 4-5 4-5 Seizure Scores
anti-GIRK1 Nterm
250
150
100
75
50
37
*
Full length
Cleaved
anti-GIRK2 Nterm
250
100
75
50
37
25
Full length
Cleaved
Short exposure
anti-GIRK2 Nterm
250
100
75
50
37
25
Full length
Cleaved
Long exposure
anti-c-Fos
250
100
75
50
37
25
*
c-Fos
*
anti-GAPDH
37
25
20
15
GAPDH

Supplementary Figure S8. Kainate-induced status epilepticus in mice induces C-terminal cleavage of GIRK2 in their hippocampi. (A) Onset of behavioral hyperactivity within 20 min and Racine scale stage 3-4 seizures and 4-5 seizures in male mice was observed after i.p injection of kainate (15 and 30 mg/kg, respectively) but not vehicle control (saline). (B-D) Immunoblotting of hippocampal membrane and soluble fractions from male mice at 8 h after injection with kainate or vehicle control (H_2O) using antibodies (all from Cell Signaling) against GIRK2 N-terminus (B), GAPDH (loading control) (C), c-Jun and c-Fos (positive control for seizures) (D). Immunoblot for PSD-95 and tubulin showed successful fractionations of soluble S2 and membrane P2 fractions. * points at non-specific bands.

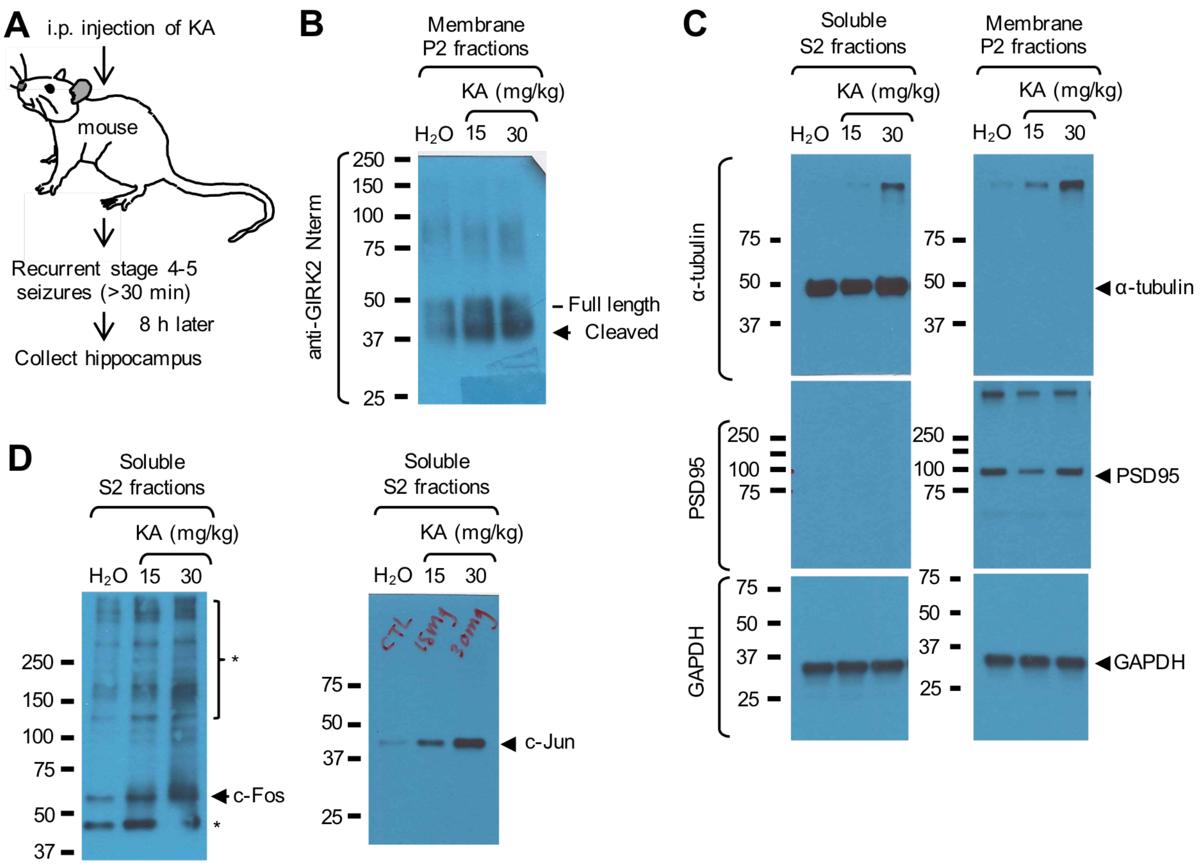

Supplement: Supplementary file 1 — Supplementary Information [file 41598_2017_12508_MOESM1_ESM.pdf]
